# Multiple health behaviour change primary care intervention for smoking cessation, physical activity and healthy diet in adults 45 to 75 years old (EIRA study): a hybrid effectiveness-implementation cluster randomised trial

**DOI:** 10.1186/s12889-021-11982-4

**Published:** 2021-12-04

**Authors:** Edurne Zabaleta-del-Olmo, Marc Casajuana-Closas, Tomàs López-Jiménez, Haizea Pombo, Mariona Pons-Vigués, Enriqueta Pujol-Ribera, Carmen Cabezas-Peña, Joan Llobera, Ruth Martí-Lluch, Caterina Vicens, Emma Motrico, Irene Gómez-Gómez, José-Ángel Maderuelo-Fernández, José I. Recio-Rodriguez, Barbara Masluk, Sara Contreras-Martos, Constanza Jacques-Aviñó, Ignacio Aznar-Lou, Montserrat Gil-Girbau, Ana Clavería, Rosa Magallón-Botaya, Juan-Ángel Bellón, Rafel Ramos, Alvaro Sanchez-Perez, Patricia Moreno-Peral, Alfonso Leiva, Clara González-Formoso, Bonaventura Bolíbar

**Affiliations:** 1 Fundació Institut Universitari per a la recerca a l’Atenció Primària de Salut Jordi Gol i Gurina (IDIAPJGol), Gran Via de les Corts Catalanes 587, 08007 Barcelona, Spain; 2 Gerència Territorial de Barcelona, Institut Català de la Salut, Balmes 22, 08007 Barcelona, Spain; 3 Nursing Department, Nursing Faculty, Universitat de Girona, Emili Grahit 77, 17003 Girona, Spain; 4 Universitat Autònoma de Barcelona, Cerdanyola del Vallès, 08193 Bellaterra, Spain; 5 Ezkerraldea-Enkarterri-Cruces Integrated Health Organisation-Biocruces Bizkaia Health Research Institute Innovation Unit, Plaza de Cruces s/n, 48903 Barakaldo, Bizkaia Spain; 6 Deputy Directorate of Healthcare Assistance, Osakidetza-Servicio Vasco de Salud, C/ Araba 45, 01006 Vitoria, Araba Spain; 7 Àrea Assistencial. Servei Català de la Salut (CatSalut), Travessera de les Corts 131-159, Edifici Olímpia, 08228 Barcelona, Spain; 8 Department of Health, Deputy Directorate of Health Promotion, Public Health Agency, Goverment of Catalonia, Roc Boronat, 81-95 (Edifici Salvany), 08005 Barcelona, Spain; 9 Unitat de Recerca, Atenció Primaria de Mallorca, Servei de Salut de les Illes Balears, C/Escola Graduada 3, 07002 Palma, Spain; 10 Institut de Investigació Sanitària de les Illes Balears (IdISBa), Carretera de Valldemossa, 79. Hospital Universitari Son Espases, Ed S., 070112 Palma, Spain; 11 Unitat de suport a la recerca de Girona. Fundació Institut Universitari per a la recerca a l’Atenció Primària de Salut Jordi Gol i Gurina (IDIAPJGol), Carrer Maluquer Salvador 11, 17002 Girona, Spain; 12 Group of research in Vascular Health, Girona Biomedical Research Institute (IdibGi), Parc Hospitalari Martí Julià - Edifici M2, Carrer del Dr. Castany, s/n, 17190 Salt, Spain; 13 Centro de Salud Son Serra-La Vileta (Ibsalut), Masanella 22, 07013 Palma, Balearic Islands Spain; 14 Facultat de Medicina. Universitat de les Illes Balears, Carretera de Valldemossa, km 7.5, 07122 Palma, Balearic Islands Spain; 15 Universidad Loyola Andalucía, Avda. de las Universidades, s/n, 41704 Dos Hermanas, Sevilla Spain; 16 Institute of Biomedical Research of Salamanca (IBSAL), Edificio Virgen de la Vega, 10.a planta. Paseo de San Vicente, 58-182, 37007 Salamanca, Spain; 17 Unidad de Investigación de Atención Primaria de Salamanca (APISAL), Instituto de Investigación Biomédica de Salamanca (IBSAL), Avda. Portugal 83, 37005 Salamanca, Spain; 18 Health Service of Castilla y León (SACyL), C/ Arapiles, 25 – 33, 37007 Salamanca, Spain; 19 Departamento de Enfermería y Fisioterapia, Universidad de Salamanca, Calle Donantes de Sangre, s/n, 37007 Salamanca, Spain; 20 Departamento de Psicología y Sociología, Universidad de Zaragoza, C/Pedro Cerbuna 12, 50009 Zaragoza, Spain; 21 Grupo Aragonés de Investigación en Atención Primaria (GAIAP), Instituto de Investigación Sanitaria, Avda. San Juan Bosco 13, 50009 Zaragoza, Spain; 22 Research and Development Unit, Institut de Recerca Sant Joan de Déu, C\ Doctor Antoni Pujadas 42, 08830 Sant Boi de Llobregat, Spain; 23 Consortium for Biomedical Research in Epidemiology & Public Health (CIBER en Epidemiología y Salud Pública—CIBERESP), 28029 Madrid, Spain; 24 Área de Xestión Integrada de Vigo, Servizio Galego de Saúde, c/Rosalía Castro 21-23, 36201 Vigo, Spain; 25 Instituto de Investigación Sanitaria Galicia Sur, Hospital Álvaro Cunqueiro, Bloque Técnico, Planta 2, Carretera Clara Campoamor n° 341, Beade, 36213 Vigo, Spain; 26 Facultad de Medicina, Universidad de Zaragoza, c/ Domingo Miral s/n, 50009 Zaragoza, Spain; 27 Arrabal Health Centre, Servicio Aragonés de Salud, Andador Aragüés del Puerto 3, 50015 Zaragoza, Spain; 28 Institute of health research of Aragon (IIS Aragón), Avda. San Juan Bosco, 13, 50009 Zaragoza, Spain; 29 Instituto de Investigación Biomédica de Málaga (IBIMA), Hospital Civil Pabellón 5. 2a Planta, Plaza del Hospital Civil, s/n, 29009 Málaga, Spain; 30 El Palo Health Centre’, Andalusian Health Service (SAS), Avenida Salvador Allende 159, 29018 Málaga, Spain; 31 Department of Public Health and Psychiatry, Facultad de Medicina, University of Málaga (UMA), Campus de Teatinos, 29071 Málaga, Spain; 32 Department of Medical Sciences, School of Medicine, Campus Salut, Universitat de Girona, Emili Grahit 77, 17003 Girona, Spain; 33 Primary Care Research Unit, Deputy Directorate of Healthcare Assistance- BioCruces Bizkaia Health Research Institute, Basque Healthcare Service –Osakidetza, Plaza Cruces s/n, E-48903 Barakaldo, Spain; 34 Unidade de Calidade de Coidados, Área sanitaria de Vigo. Hospital Álvaro Cunqueiro, Estrada Clara Campoamor n° 341, 36312 Vigo, Spain

**Keywords:** Health behaviour, Health promotion, Hybrid trial, Implementation science, Mediterranean diet, Physical activity, Primary health care, Smoking cessation

## Abstract

**Background:**

This study aimed to evaluate the effectiveness of a) a Multiple Health Behaviour Change (MHBC) intervention on reducing smoking, increasing physical activity and adherence to a Mediterranean dietary pattern in people aged 45–75 years compared to usual care; and b) an implementation strategy.

**Methods:**

A cluster randomised effectiveness-implementation hybrid trial-type 2 with two parallel groups was conducted in 25 Spanish Primary Health Care (PHC) centres (3062 participants): 12 centres (1481 participants) were randomised to the intervention and 13 (1581 participants) to the control group (usual care). The intervention was based on the Transtheoretical Model and focused on all target behaviours using individual, group and community approaches. PHC professionals made it during routine care. The implementation strategy was based on the Consolidated Framework for Implementation Research (CFIR). Data were analysed using generalised linear mixed models, accounting for clustering. A mixed-methods data analysis was used to evaluate implementation outcomes (adoption, acceptability, appropriateness, feasibility and fidelity) and determinants of implementation success.

**Results:**

14.5% of participants in the intervention group and 8.9% in the usual care group showed a positive change in two or all the target behaviours. Intervention was more effective in promoting dietary behaviour change (31.9% vs 21.4%). The overall adoption rate by professionals was 48.7%. Early and final appropriateness were perceived by professionals as moderate. Early acceptability was high, whereas final acceptability was only moderate. Initial and final acceptability as perceived by the participants was high, and appropriateness moderate. Consent and recruitment rates were 82.0% and 65.5%, respectively, intervention uptake was 89.5% and completion rate 74.7%. The global value of the percentage of approaches with fidelity ≥50% was 16.7%. Eight CFIR constructs distinguished between high and low implementation, five corresponding to the *Inner Setting* domain.

**Conclusions:**

Compared to usual care, the EIRA intervention was more effective in promoting MHBC and dietary behaviour change. Implementation outcomes were satisfactory except for the fidelity to the planned intervention, which was low. The organisational and structural contexts of the centres proved to be significant determinants of implementation effectiveness.

**Trial registration:**

ClinicalTrials.gov, NCT03136211. Registered 2 May 2017, “retrospectively registered”.

**Supplementary Information:**

The online version contains supplementary material available at 10.1186/s12889-021-11982-4.

## Background

In 2016, 71% of global deaths were due to non-communicable diseases (NCDs) such as heart disease, stroke, cancer, chronic respiratory diseases and diabetes [1]. Smoking, insufficient physical activity, and unhealthy diet are three modifiable health behaviours that underlie most of these conditions [2]. Consequently, focusing on shifting these behaviours might significantly strengthen the prevention and control of NCDs [3]. Health promotion interventions usually focus on a single health behaviour change (BC); however, adults often engage in two or more unhealthy behaviours simultaneously. Various studies show that in adults, the co-occurrence of unhealthy diet with insufficient physical activity ranges between 47 and 54%, unhealthy diet with smoking between 23 and 28%, and insufficient physical activity with smoking between 8 and 20% [4]. Furthermore, the co-occurrence of more than one unhealthy behaviour has an additive and even synergistic negative impact on health [5]. Accordingly, Multiple Health Behaviour Change (MHBC), efforts to treat two or more health behaviours, seems the logical choice for improving people’s lifestyles and health. Notably, while MHBC interventions have produced a modest reduction in unhealthy behaviours [6], studies show that small lifestyle changes might have considerable and sustained benefits on people’s health and quality of life [3, 7].

In addition, multiple unhealthy behaviours are closely associated with socioeconomic factors and health inequalities [4, 6]. Consequently, awareness of motivations, opportunities, capacities and social and physical environments are crucial to successful MHBC interventions [8]. In this regard, Primary Health Care (PHC) is considered the most convenient setting to promote BC since it is highly accessible, has an integral approach to health and provides continuity of care [3, 9]. However, the implementation of health promotion and prevention interventions in PHC remains suboptimal, mainly due to work overload and lack of time or training [10, 11]. In addition to all these barriers, the most suitable model to approach BC remains unclear, and there is a lack of theoretical basis of interventions, skills in helping people changing behaviour and knowledge of the local context in which these interventions are undertaken [12–14].

To incorporate all this complexity, the Medical Research Council (MRC) proposed a methodology that promotes the participation of citizens and professionals in research, thus increasing the acceptability and feasibility of interventions [15–17]. This methodology also considers the sustainability of interventions and the transfer of research to PHC practice [16]. Thus, it represents a turning point in the conventional way of conducting experimental studies in which the most important thing is finding value and understanding the context of practice rather than trying to control its influence [15, 17, 18].

Furthermore, research on MHBC interventions in PHC should not only determine their effectiveness but also provide evidence on the most successful strategies for implementation in real-world settings [19]. Central to this is the field of implementation research whose approach and the subject of study are aligned with the MRC framework [15–17]. Implementation research provides evidence on a comprehensive set of research questions, ranging from implementation outcomes to implementation determinants or identifying the most successful implementation strategies [20]. Regarding this, the effectiveness-implementation hybrid trials with their dual approach offer the opportunity to assess the effectiveness of both an intervention and an implementation strategy [20, 21].

Therefore, in 2012, the Spanish Primary Care Prevention and Health Promotion Research Network (redIAPP) [22] launched the EIRA study, a MHBC intervention targeting three unhealthy behaviours in people aged 45 to 75. The first three phases (preclinical phase, phase I and phase II) followed the MRC framework [11, 18, 23–31]. This article describes the results of phase III, in which we used a hybrid design to evaluate the effectiveness of a) a 12-month MHBC primary care intervention on reducing smoking, increasing physical activity, and enhancing adherence to a Mediterranean dietary pattern in people aged 45–75 years compared to usual care; and b) an implementation strategy in terms of acceptability, adoption, appropriateness, feasibility and fidelity.

## Methods

### Study design

The study was a cluster randomised effectiveness-implementation hybrid trial-type 2 with two parallel groups. Results are reported according to the Standards for Reporting Implementation Studies (StaRI) [32] and Consolidated Standards of Reporting Trials (CONSORT) statement for the reporting of cluster randomised trials [33]. Details of the study rationale and design of the study as well as the economic evaluation of the intervention have been previously published [34, 35].

### Context

The study was conducted from January 2017 to December 2018 in PHC centres of seven of the 17 Spanish Autonomous Communities. The Spanish National Health System has universal coverage with free access to health care for the entire population, public financing, integration of different health service networks, and a gatekeeping system at PHC level. PHC includes health care, health education and prevention, health promotion and community care and is provided by multidisciplinary teams (physicians, nurses, paediatricians, social workers, and dentists) in a defined population area.

### Targeted sites and populations

EIRA study comprised two targets: PHC centres and PHC users [35].

Twenty-six PHC centres participated. To be enrolled in the study, PHC centres had to have internet access, be able to implement community activities, not be located in culturally and linguistically diverse or tourist areas and have a pro-actively engaged management team. All healthcare and administrative staff were invited to participate.

PHC users were people aged 45 to 75 years who engaged in at least two of the three following unhealthy behaviours: smoking, insufficient physical activity, and low adherence to a Mediterranean dietary pattern. PHC users with advanced serious illness, cognitive impairment, dependence for basic activities of daily living, severe mental illness, and those in a long-term home health care programme, undergoing cancer treatment or end-of-life care or those planning to move from the area during the intervention were excluded.

### Intervention

**Fig. 1:**
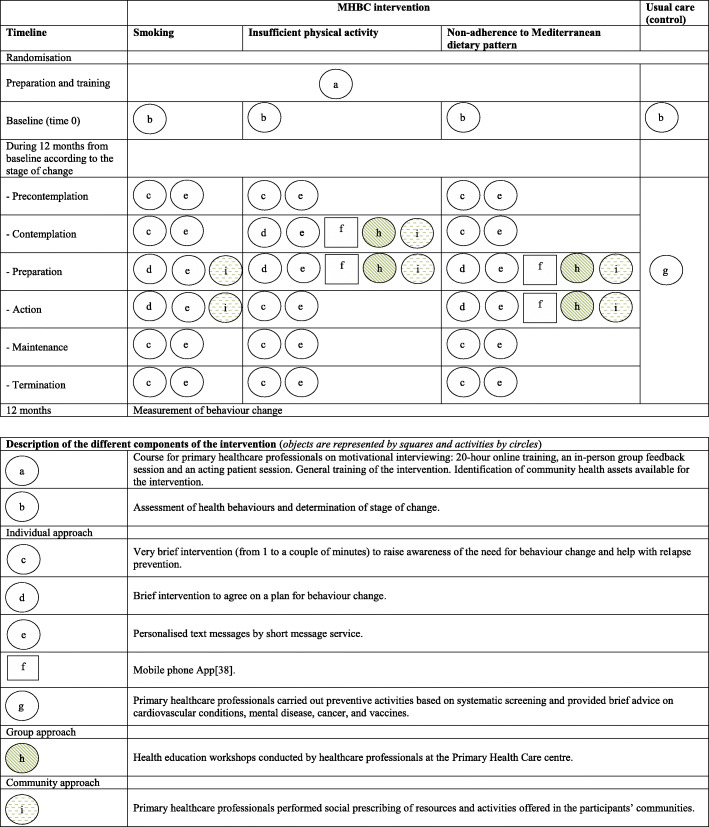
Graphical depiction of intervention based on the proposals by Perera et al. [39]

The intervention was based on the Transtheoretical Model (TTM) and Stages of Change [24, 36] and integrated into the daily practice of PHC professionals. It consisted of a first screening visit in which the PHC professionals assessed target behaviours and stages of change [35]. For behavioural screening, we used one question on tobacco use during the previous month, two validated questions to estimate the daily number of servings of fruit and vegetables [37], and the *Brief Physical Activity Assessment Tool* [38]. The intervention was built on the results of the previous phases of the EIRA study [11, 18, 23–31], had a maximum duration of 12 months and was carried out at the individual, group and community level in accordance with the stages of change and unhealthy behaviours (see Fig. 1). The intervention focused on all target behaviours, and together, participant and PHC professional developed priority actions on one or more of these behaviours.

The individual approach [35] had an average intensity of 2–3 visits; the professionals could add extra visits when appropriate. Depending on the stages of change, the visit included: a) a very brief intervention to raise awareness of the need for MHBC and help with relapse prevention; b) a brief intervention to agree on a plan for MHBC. Health professionals enhanced their motivational interviewing skills with a 20-h online training, an in-person group feedback session and an acting patient session. In addition, PHC professionals and participants benefited from web-based tools such as http://proyectoeira.rediapp.org, personalised text messages, and a mobile app [40]. In addition, if participants had their own pedometers and smartwatches, advice and guidance on their use was given.

The group approach [35] consisted of health education workshops on healthy diet and physical activity, delivered some weeks after initiating the individual approach and were conducted by healthcare professionals at the PHC centre. These workshops lasted 90–120 min. Their primary purpose was to strengthen the advice discussed during the individual visits and provide people with guidelines toward practising physical activity and adopting a healthy diet, for example, through gym sessions, cooking workshops, and seasonal menus.

The community approach [35] focused mainly on social prescription of resources and activities offered in the participants’ communities. Previously, the PHC teams identified the community health assets and selected the most relevant, accessibility and possibility of referral of participants. These community activities included cooking courses, healthy eating workshops, local walking events, line dances and other physical activity programs.

### Usual care

PHC professionals in the control group integrated a Program of Preventive Activities and Health Promotion [41], which incorporates preventive protocols with lifestyle recommendations and activities targeting specific age, sex and risk groups. Preventive activities were based on systematic screening, and brief advice was provided on cardiovascular conditions, mental disease, cancer, and vaccines [35].

### Implementation strategy

The implementation strategy was based on the following:

a) The Consolidated Framework for Implementation Research (CFIR) [42], which identifies five domains: intervention characteristics, outer setting, inner setting, characteristics of individuals, and the implementation process itself.

b) A set of discrete implementation strategies [43] based on planning, education, finance, restructuration, and quality management.

This implementation strategy was built on the findings of the previous phases of the EIRA study [11, 18, 23–31] carried out in three stages (pre-implementation, implementation and post-implementation) (see Table 1).

**Table 1:** Description of implementation strategies

Stage	Key element	Description
Pre-implementation	Barriers and facilitators	During this stage, the literature was reviewed. The researchers assessed local needs, resources, barriers and facilitators to develop specific implementation strategies. Perspectives of clinicians on internal resources were measured by the “Survey of Organizational Attributes for Primary Care”.
	Support materials	All the support material for the intervention was prepared.
	Management and quality control systems	Mechanisms for effective communication and the case report form were defined and piloted. A checklist (online database) was developed and piloted to monitor the implementation progress in each Primary Health Care centre.
	Facilitation and leadership	The facilitator (member of the research team) and the leader (member of the primary care team) of the implementation were designated.
	Commitment of stakeholders	Formal commitments were established with the managers (at the macro, meso and micro levels), professionals of the centres involved and community partners.
	Training	Training activities were conducted, specially training in motivational interview
	Collaborative modelling	Local sessions to adapt and tailor the intervention to the specific context through shared decision making.
Implementation	Collaborative learning	The facilitator and the implementation leader monitored implementation processes, identified opportunities for improvement and optimised implementation.
	Commitment of main stakeholders	Audit and feedback techniques were used with the main stakeholders to maintain the commitment and the motivation.
	Training	Health professionals received continuous training in motivational interview.
Post-implementation	Management and quality control systems	The implementation evaluation was conducted using qualitative and quantitative methodologies

### Recruitment

Several interactive and passive recruitment strategies were used to increase the feasibility of achieving the target sample size [44]. The most frequent strategy was the recruitment at the time of visit as part of usual care, and it was complemented with other four recruitment strategies: 1) self-administered questionnaires delivered in the waiting room or the admission desk; 2) a part-time training recruiter; 3) advertising by posters in the PHC centres and 4) phone calls to selected patients from the review of electronic health records.

### Assignment of intervention

Participating PHC professionals signed a collaboration commitment to the study before the allocation of the intervention. The PHC centres were computer randomised for the intervention at a central location (IDIAP Jordi Gol, Barcelona, Spain). In each of the seven Spanish Autonomous Communities, half of the PHC centres (*n* = 13) were allocated to the intervention and the other half (*n* = 13) to the control group. PHC professionals were aware of the study allocation. An external unit independent of the PHC centre evaluated the intervention at baseline and the end to minimise bias.

### Evaluation

#### Intervention evaluation

The effectiveness of the intervention compared to usual care at 12 months post-intervention was measured by:

- Positive change in smoking behaviour: self-reported continuous abstinence [70]. Positive change was defined as smoking at study entry and not smoking at the end of the study. We measured punctual and continuous abstinence at these two times.

- Positive change in physical activity behaviour: sufficient physical activity in previously insufficiently active people. The *International Physical Activity Questionnaire* was used [45], and participants were classified into three physical activity categories (high, moderate, and low) according to its scoring protocol [46]. Positive change was defined as having a low physical activity level at baseline and a moderate or high physical activity level at the end of the study.

- Positive change in dietary behaviour: adherence to a Mediterranean dietary pattern in people with low adherence at baseline. The 14-item Questionnaire of Mediterranean Diet Adherence (PREDIMED study) [47] was used. Positive change was defined as obtaining eight or fewer points at study entry and nine or more at the end of the study.

#### Statistical methods

A sample size of 3640 participants (1820 for each group), allowing for 30% loss to follow-up, was estimated to have 80% power (at 5% significance level, two-tailed and with an intracluster correlation of 0.01, 48] to detect an absolute difference in a positive change in one or more of the three behaviours of 8% between groups (EIRA intervention and usual care).

A statistical analysis plan was established before data were available [35]. All data were analysed on an intention-to-treat basis. We compared cluster and participant characteristics for all variables of interest by group allocation, using either means (standard deviations) or medians (interquartile ranges) for continuous variables and numbers (percentages) for categorical variables. To address potential biases due to incomplete follow-up and nonresponse in surveys, multiple imputations by chained equations (mice function in R software) with 50 imputed datasets were applied to outcomes and covariates. Estimates from each imputed dataset were combined following the rules outlined by Rubin [71]. We assumed that the missing data were Missing At Random (MAR). The MAR assumption becomes more plausible by collecting more explanatory variables and including them in the analysis. Therefore, we included most possible explanatory variables (excluding duplicate, very similar, and highly correlated variables to avoid collinearity) [49].

To analyse the effect of the intervention on each outcome measure, Odds Ratios (OR) and their 95% confidence intervals were computed by logistic regression models for clustered data, specifically generalised linear mixed models (using Stata function xtmelogit) with the PHC centre as a random-effects parameter. We analysed the variables associated with smoking cessation, the change in physical activity and adherence to a Mediterranean dietary pattern, as well as the change in any behaviour and two or three behaviours, adjusting for possible confounding variables. Final models were chosen in accordance with the study objectives, prior research [10, 11] and the nature of the variables (potential confounders, significant and clinically relevant variables). We also calculated an overall impact factor of the intervention on the target population according to an expanded impact formula for MHBC proposed by Prochaska et al. [50]: ∑# of behaviours (n) (E_n_ x P_n_), where P is the proportion of the sample at risk for each behaviour and E is the estimate of efficacy for each behaviour. We used Stata/SE v.15.1 (StataCorp, LP, TX) and SPSS 25.0 (SPSS Inc., Chicago, Illinois) for all analyses.

#### Implementation evaluation

We assessed implementation outcomes and the determinants of implementation success.

#### Implementation outcomes

The following implementation outcomes based on the evaluation framework proposed by Proctor et al. [51] were assessed:

#### Adoption

We calculated the proportion of PHC professionals who pre-implementation indicated their intention to implement the EIRA intervention.

#### *Appropriateness and acceptability* (early and final)

Both implementation outcomes were assessed on PHC professionals and participants. *Appropriateness* was defined as the perceived fit or relevance of the intervention. Related terms were relevance, perceived fit, compatibility, trialability, suitability, usefulness, and practicability. *Acceptability* was defined as the perception that the intervention was agreeable, and related terms were content, complexity, comfort, relative advantage, and credibility. We designed two self-administered questionnaires, one for participants and one for PHC professionals. Two instruments were administered in the pre and post-implementation stages. The definitions of implementation outcomes [51], related terms [52] and other measurement instruments available [53] constituted the conceptual model to define the items. A set of potentially relevant items was formulated. Questionnaires were pilot-tested in phase II of the study. The final questionnaire for PHC professionals included eight items, and the participants’ questionnaire included seven. All items in both questionnaires used an 11-point Likert scale with three semantic anchors. In the questionnaire for professionals, the appropriateness and acceptability of the intervention were measured according to the type of unhealthy behaviour. In contrast, the items were more generic in the participants’ questionnaire. Supplementary file 1 includes a copy of both questionnaires. We analysed the structure of questionnaires, and factorial analysis found two dimensions in both questionnaires. Goodness-of-fit indices suggested a good model fit in the professionals’ questionnaire (Root Mean Square Error of Approximation (RMSEA) = 0.05 and Comparative Fit Index (CFI) = 0.99) and adequate fit in the participants’ questionnaire (RMSEA = 0.06 and CFI = 0.99). Similarly, internal consistency in the scores of the two dimensions was good in both questionnaires (Cronbach’s alpha ≥0.80).

#### Feasibility

We calculated consent rate (% participants who consented among all invited to participate), recruitment rate (% of participants who were eligible, who accepted and attended the baseline assessment visit among all those invited to participate), intervention uptake rate (% of recruited participants who received the intervention) and completion rate (% of recruited participants who completed the study).

#### Fidelity of the planned intervention

The degree of compliance with planned activities for each intervention approach was estimated by analysing the number and kind of activities recorded in the case report form (CRF) by PHC professionals.

**Determinants of implementation success.** Ten focus group meetings moderated by an experienced researcher were conducted in the post-implementation stage. A total of 64 PHC professionals (average number per group = 8) from intervention centres participated. We were unable to perform this evaluation in two PHC centres (PHC centres C and E) due to major staff and management team changes where PHC team members were not the same as those who participated in the EIRA study at the time. A structured interview guide based on CFIR constructs was used [54]. We used the CFIR tool (available from https://cfirguide.org/guide/app/#/) to create this interview guide. Some examples of the questions used can be consulted in Supplementary file 2. Group sessions were recorded and transcribed to create written documents for qualitative coding. All transcripts were reviewed by the research team members (BB, CJA, EZO, HP, MCC and TLJ). Thematic content analysis and data coding were performed in accordance with CFIR constructs. Coding was deductive (codes derived from CFIR constructs) and inductive (codes derived from the data). Subsequently, researchers rated each CFIR construct for each PHC centre according to CFIR guidelines. Ratings ranged from − 2 to + 2, with 0 representing a neutral or mixed influence and M representing missing data. Two researchers independently coded and rated data of each PHC centre and wrote a memo report which was subsequently discussed with the whole team of analysts until an agreement was reached. During all this analysis, researchers were blinded to the intervention and implementation outcomes.

Spearman’s rank correlation coefficients were computed and used to assess the strength of correlation between construct ratings and fidelity of the planned intervention across PHC centres. Constructs with statistically significant correlations (*P* < 0.05) with *fidelity* outcomes were believed to strongly distinguish PHC centres with low and high implementation success. Correlations values of rho ≥0.50, but with *P* values between 0.05 and 0.10 were considered weakly distinguishing.

### Patient and public involvement

Patients were involved in the design, recruitment and development of the study in agreement with the methodology proposed by the MRC [23, 25, 27]. Patients also communicated their perception of the appropriateness and acceptability of the intervention. Once the study is published, participants will be informed of the results via the websites www.idiapjgol.organd www.rediapp.org, and the Twitter account @IDIAPJGol. Press releases aimed at a lay public will also be published.

## Results

### Proportion recruited and characteristics

**Fig. 2:**
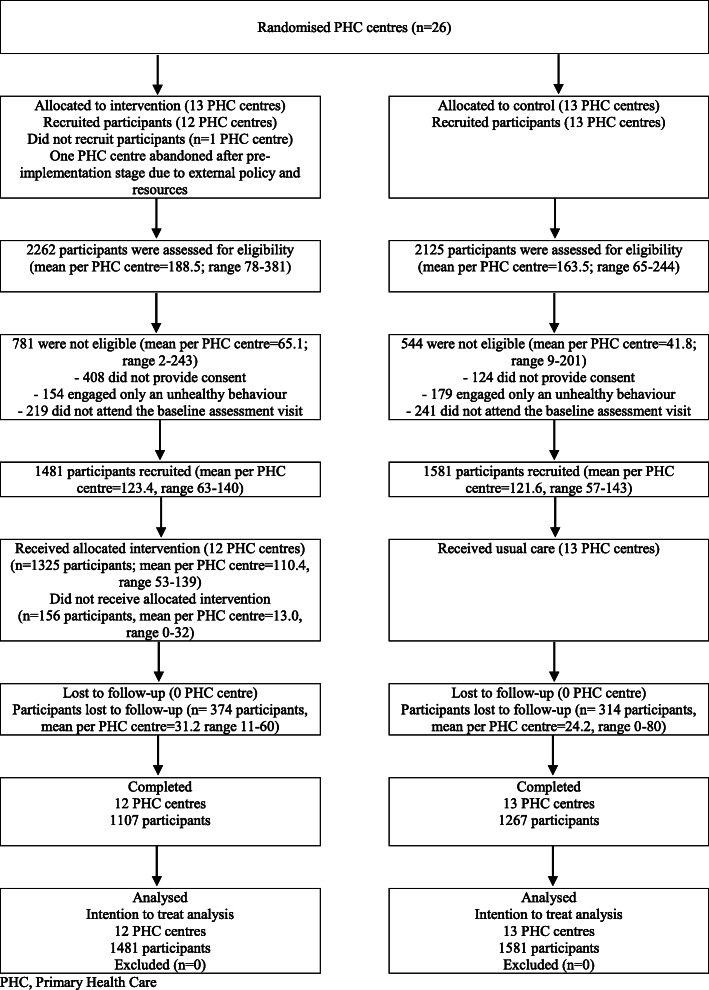
Flow diagram of clusters and participants

One PHC centre dropped out of the intervention group after the pre-implementation stage due to unfavourable external policy and lack of available resources. In total, 3062 participants from 25 PHC centres were recruited, 1481 participants in 12 intervention centres and 1581 in 13 control centres. The percentage of losses during follow-up was 25.3 and 19.9% in the intervention and control groups, respectively. The flowchart of participants is presented in Fig. 2. PHC centres and participants were similar at baseline, except for some differences at cluster level (higher percentage of PHC training centres in the control group), and at participant level (higher percentage of people in a state of precontemplation in the control PHC centres and a state of preparation in the intervention PHC centres for all three unhealthy behaviours) (see Table 2).

**Table 2:** Baseline characteristics at cluster and individual levels of randomised groups: usual care (control) and EIRA intervention

	Usual care	EIRA Intervention	Total	
**Cluster level (PHC centre)**				
**Characteristics of the PHC centres**	***n*** **= 13**	***n*** **= 12**	***n*** **= 25**	
Population assigned, mean (SD)	21,816 (13974)	22,881 (7662)	22,327 (11173)	
Population age, mean (SD)	44.5 (4.9)	42.0 (4.0)	43.3 (4.6)	
% immigrant population, mean (SD)	12.6 (10.9)	11.8 (9.1)	12.2 (9.9)	
Number of healthcare professionals working in the PHC centre, mean (SD)				
Physicians	14.7 (7.9)	12.2 (5.3)	13.5 (6.8)	
Nurses	13.3 (9.5)	12.2 (5.6)	12.8 (7.8)	
Training centre for PHC professionals, n(%)	11 (84.6)	8 (66.7)	19 (76.0)	
**Characteristics of professionals who participated in the survey**	***n*** **= 208**	***n*** **= 223**	***n*** **= 431**	
Age (years), mean (SD)	49.9 (11.20)	48.8 (11.15)	49.3 (11.17)	
Time (years) working in PHC, mean (SD)	19.1 (11.98)	18.3 (11.16)	18.7 (11.55)	
Time (years) working in the same PHC centre, mean (SD)	9.8 (8.44)	9.4 (8.35)	9.6 (8.38)	
Female, n (%)	165 (79.3)	179 (80.3)	344 (79.8)	
*Academic training, n (%)*				
- Master’s or PhD training	27 (13.0)	42 (18.8)	69 (16.1)	
*Healthy behaviours, n (%)*				
- Non-smokers/Ex-smokers	175 (84.1)	165 (74.0)	340 (78.8)	
- Sufficiently active	129 (62.0)	122 (54.7)	251 (58.2)	
- Mediterranean diet adherent	119 (57.2)	121 (54,3)	240 (55.7)	
Survey of Organizational Attributes for Primary Care, mean (SD)*	***n*** **= 185**	***n*** **= 182**	***n*** **= 367**	
- Communication	6.6 (1.7)	6.5 (1.7)	6.5 (1.7)	
- Practice-wide decision-making	6.6 (1.8)	6.3 (2.0)	6.4 (1.9)	
- Nurses’ participation in decision-making	7.0 (1.9)	6.6 (2.2)	6.8 (2.1)	
- Stress/chaos	5.3 (1.8)	4.7 (1.8)	5.0 (1.8)	
- History of change	5.5 (2.1)	5.1 (2.0)	5.3 (2.1)	
**Individual level (participants)**				
**Characteristics of participants**	***n*** **= 1581**	***n*** **= 1481**	***n*** **= 3062**	**% missing values**
Age (years), median (IQR)	57 (51–65)	57 (51–64)	57 (51–65)	0.0
45–54 years	612 (38.7)	574 (38.8)	1186 (38.7)	
55–64 years	519 (32.8)	558 (37.7)	1077 (35.2)	
65–75 years	450 (28.5)	349 (23.6)	799 (26.1)	
Female, n (%)	872 (55.2)	809 (54.6)	1681 (54.9)	0.0
Smokers, n(%)	697 (44.1)	638 (43.1)	1335 (43.6)	0.0
Insufficiently active, n (%)	1448 (91.6)	1345 (90.8)	2793 (91.2)	0.0
Non-adherent Mediterranean dietary pattern, n (%)	1482 (93.7)	1384 (93.5)	2866 (93.6)	0.0
Co-occurrence of unhealthy behaviours				0.0
Non-adherence to Mediterranean dietary pattern & Insufficient physical activity	884 (55.9)	843 (56.9)	1727 (56.4)	
Non-adherence to Mediterranean dietary pattern & Smoking	133 (8.4)	136 (9.2)	269 (8.8)	
Smoking & Insufficient physical activity	99 (6.3)	97 (6.5)	196 (6.4)	
Non-adherence to Mediterranean dietary pattern & Insufficient physical activity & Smoking	465 (29.4)	405 (27.3)	870 (28.4)	
**Stage of change: smoking, n (%)**				
Precontemplation	398 (25.2)	168 (11.3)	566 (18.5)	4.1
Contemplation	196 (12.4)	163 (11.0)	359 (11.7)	
Preparation	66 (4.2)	170 (11.5)	236 (7.7)	
Action	13 (0.8)	35 (2.4)	48 (1.6)	
Maintenance/Termination	885 (56.0)	841 (56.8)	1726 (56.4)	
**Stage of change: physical activity, n (%)**				
Precontemplation	643 (40.7%)	152 (10.3)	795 (26.0)	8.0
Contemplation	389 (24.6)	229 (15.5)	618 (20.2)	
Preparation	204 (12.9)	516 (34.8)	720 (23.5)	
Action	137 (8.7)	266 (18.0)	403 (13.2)	
Maintenance/Termination	175 (11.1)	107 (7.2)	282 (9.2)	
**Stage of change: Mediterranean dietary pattern, n (%)**				
Precontemplation	554 (35.0)	98 (6.6)	652 (21.3)	7.8
Contemplation	295 (18.7)	220 (14.9)	515 (16.8)	
Preparation	206 (13.0)	619 (41.8)	825 (26.9)	
Action	179 (11.3)	244 (16.5)	423 (13.8)	
Maintenance/Termination	318 (20.1)	89 (6.0)	407 (13.3)	
**Stages of change: Pre-action in any behaviour**	1442 (91.2%)	1135 (76.6%)	2577 (84.1%)	7.9
**Country of origin, n (%)**				
Spain	1479 (93.5)	1369 (92.4)	2848 (93.0)	0.9
Other countries	97 (6.1)	89 (6.0)	186 (6.1)	
**Education level, n (%)**				
Secondary or higher	888 (56.2)	820 (55.4)	1708 (55.8)	0.9
Primary or lower	684 (43.3)	641 (43.3)	1325 (43.3)	
**Employment status, n (%)**				
Employed	713 (45.1)	661 (44.6)	1374 (44.9)	1.0
Unemployed	141 (8.9)	145 (9.8)	286 (9.3)	
Homemaker	194 (12.3)	174 (11.7)	368 (12.0)	
Retired	431 (27.3)	371 (25.1)	802 (26.2)	
Other (student or incapacity for work)	96 (6.1)	105 (7.1)	201 (6.6)	
**Civil Status, n (%)**				
Married or cohabiting	1055 (66.7)	1024 (69.1)	2079 (67.9)	0.8
Unmarried or single	520 (32.9)	437 (29.5)	957 (31.3)	
**Total cholesterol (mg/dl), median (IQR)**	206 (179–232)	203 (178–232)	204 (178–232)	8.2
**LDL cholesterol (mg/dl), median (IQR)**	126 (105–150)	124 (101–151)	125 (103–150)	17.2
**HDL cholesterol (mg/dl), median (IQR)**	52 (43–62)	50 (43–60)	51 (43–61)	13.3
**BMI (kg/m**^**2**^**), median (IQR)**	28.4 (25.3–32.4)	30.1 (24.5–34.0)	29.4 (25.8–33.2)	1.1
**COPD, n (%)**	60 (3.8)	65 (4.4)	125 (4.1)	0.8
**Hypertension, n (%)**	610 (38.6)	587 (39.6)	1197 (39.1)	0.8
**Diabetes, n(%)**	323 (20.4)	277 (18.7)	600 (19.6)	0.5
**Health-related quality of life by the EQ-5D index, median (IQR)**	0.80 (0.71–1.00)	0.79 (0.70–1.00)	0.79 (0.70–1.00)	1.5

SD, standard deviation, IQR, interquartile range; LDL, low-density lipoprotein cholesterol; HDL, high-density lipoprotein; BMI, Body mass index; COPD, Chronic Obstructive Pulmonary Disease

*scores were standardised at 10 points, the higher the score the higher the characteristic grade, except in “stress/chaos” where the higher the score the lower the grade

### Intervention outcomes

Table 3 shows the results of the intervention outcomes. Positive changes in two or three behaviours (OR 1.70; CI 95% 1.09 to 2.63) and in any of three behaviours (OR 1.48; CI 95% 1.05 to 2.10) were significantly greater in the intervention group compared with the usual care group. Furthermore, the overall impact on participants was 0.66 in the intervention group and 0.50 in the usual care group. Of the three behaviours, the intervention was more effective than usual care in promoting dietary BC (OR 1.87; CI 95% 1.30 to 2.68). Multivariate models adjusted by covariates are described in Table 4. At the cluster level, the PHC training centre had a positive effect on dietary BC and a negative effect on physical activity BC. At the participant level, being in the preparation stage showed a positive effect on change in all three target behaviours, while being in action stages showed a positive effect on smoking cessation. Moreover, co-occurrence of non-adherence to a Mediterranean dietary pattern and insufficient physical activity was positively associated with dietary BC but negatively associated with MHBC.

**Table 3:** Effect of the EIRA intervention or usual care on positive change and quantification of overall impact of intervention

Outcomes		EIRA intervention, n/N (%)	Usual care, n/N (%)	NNC (95% CI)	Unadjusted OR (95% CI)	*P* value	Adjusted* OR (95% CI)	*P* value	Adjusted† OR (95% CI)	*P* value
1	Positive change in two or three behaviours	215/1481 (14.5)	141/1581 (8.9)	18 (12.7 to 30.1)	1.75 (1.30 to 2.36)	< 0.001	1.82 (1.19 to 2.77)	0.005	1.70 (1.09 to 2.63)	0.018
2	Positive change in **any** behaviour	810/1481 (54.7)	693/1581 (43.8)	10 (7.0 to 13.6)	1.55 (1.32 to 1.81)	< 0.001	1.60 (1.14 to 2.25)	0.006	1.48 (1.05 to 2.10)	0.027
3	Positive change in **smoking** behaviour	158/638 (24.8)	126/697 (18.1)	15 (9.0 to 43.8)	1.40 (1.04 to 1.89)	0.028	1.37 (0.93 to 2.01)	0.111	1.30 (0.89 to 2.03)	0.165
4	Positive change in **physical activity** behaviour	372/1345 (27.7)	351/1448 (24.2)	30 (15.0 to 609.9)	1.17 (0.98 to 1.41)	0.088	1.23 (0.79 to 1.92)	0.362	1.07 (0.74 to 1.54)	0.737
5	Positive change in **diet** behaviour	442/1384 (31.9)	317/1482 (21.4)	10 (7.3 to 13.7)	1.75 (1.45 to 2.11)	< 0.001	1.83 (1.26 to 2.66)	0.002	1.87 (1.30 to 2.68)	0.001

NNC, Number needed for change; CI, Confidence interval; OR, Odds Ratio;

*Adjusted for cluster effect. Intracluster correlation coefficient was for outcome 1, 0.040 (95% CI 0.016 to 0.094); for outcome 2, 0.042 (95% CI 0.021 to 0.080); for outcome 3, 0.027 (95% CI 0.009 to 0.087); for outcome 4, 0.072 (95% CI 0.038 to 0.132), and for outcome 5, 0.047 (95% CI 0.024 to 0.093)

†Adjusted for cluster effect and variables at participant level and at cluster level: *average age of health care professionals, average time (years) working in the same PHC centre, and PHC training centre (yes/no)* and variables at participant level: *baseline stage of change, age, and sex, co-occurrence of unhealthy behaviours, education level, employment situation of participant*

Overall impact factor [51]

**Table:** 

**Target behaviour**	**Proportion at risk**^**a**^	**Efficacy EIRA intervention or usual care**^**b**^	**Individual impact factor**^**c**^	**Impact factor on participants**^**d**^	**Impact factor on the population**^**e**^
**EIRA intervention**					
Smoking	0.431	0.248	0.11	0.66	0.43
Insufficient physical activity	0.908	0.277	0.25		
Non-adherence to Mediterranean dietary pattern	0.935	0.319	0.30		
**Usual care**					
Smoking	0.441	0.181	0.08	0.50	0.37
Insufficient physical activity	0.916	0.242	0.22		
Non-adherence to Mediterranean dietary pattern	0.937	0.214	0.20

^a^Proportion of the target behaviour in the study sample at baseline

^b^Proportion of participants who reached a positive change for each behaviour

^c^Proportion at risk (a) multiplied by the efficacy of the EIRA intervention or usual care (b)

^d^Sum of individual impact factors

^e^Impact factor on participants (d) multiplied by recruitment rate (0.655 in intervention centres and 0.744 in control centres)

**Table 4:** Description of multivariable models

Target behaviours	Smoking		Insufficient physical activity		Non-adherence to Mediterranean dietary pattern		Any of the three behaviours*		Two or three behaviours*	
	OR (95% CI)	P value	OR (95% CI)	P value	OR (95% CI)	P value	OR (95% CI)	P value	OR (95% CI)	P value
**Outcomes**										
Positive change in **two or three** behaviours									1.70 (1.09 to 2.63)	0.018
Positive change in **any** behaviour							1.48 (1.05 to 2.10)	0.027		
Positive change in **smoking** behaviour	1.30 (0.89 to 2.03)	0.165								
Positive change in **physical activity** behaviour			1.07 (0.74 to 1.54)	0.737						
Positive change in **diet** behaviour					1.87 (1.30 to 2.68)	0.001				
**Variables at cluster level**										
Average age of healthcare professionals	0.99 (0.96 to 1.03)	0.727	1.01 (0.98 to 1.05)	0.545	0.99 (0.96 to 1.02)	0.452	1.00 (0.96 to 1.03)	0.788	1.00 (0.96 to 1.04)	0.833
Average time (years) working in the same PHC centre	0.96 (0.92 to 1.02)	0.189	0.99 (0.94 to 1.05)	0.842	0.97 (0.93 to 1.02)	0.313	0.98 (0.93 to 1.03)	0.355	0.96 (0.90 to 1.02)	0.158
Training centre for PHC professionals (yes vs. no)	1.29 (0.82 to 2.03)	0.264	0.56 (0.37 to 0.86)	0.008	1.62 (1.08 to 2.43)	0.019	0.97 (0.65 to 1.44)	0.867	1.03 (0.63 to 1.67)	0.909
**Variables at participant level**										
Age (years)	1.00 (0.97 to 1.03)	0.988	0.99 (0.98 to 1.01)	0.401	1.00 (0.99 to 1.02)	0.750	1.00 (0.99 to 1.02)	0.873	0.99 (0.97 to 1.02)	0.616
Female vs. male	0.88 (0.61 to 1.26)	0.480	1.01 (0.83 to 1.23)	0.945	1.16 (0.95 to 1.42)	0.153	1.05 (0.88 to 1.26)	0.563	1.02 (0.77 to 1.36)	0.892
Stage of change										
*- Precontemplation*	1.00		1.00		1.00		1.08 (0.87 to 1.34)	0.472	0.93 (0.64 to 1.34)	0.693
*- Contemplation*	1.09 (0.68 to 1.75)	0.723	1.19 (0.90 to 1.59)	0.229	1.21 (0.86 to 1.71)	0.282	1.16 (0.94 to 1.42)	0.168	1.29 (0.92 to 1.81)	0.137
*- Preparation*	1.57 (0.97 to 2.53)	0.065	1.26 (0.93 to 1.70)	0.134	1.13 (0.81 to 1.57)	0.487	1.30 (1.06 to 1.60)	0.013	1.19 (0.86 to 1.65)	0.285
*- Action, maintenance or termination*	2.53 (1.19 to 5.38)	0.016	0.81 (0.58 to 1.14)	0.235	1.02 (0.74 to 1.41)	0.906	1.07 (0.80 to 1.43)	0.639	0.90 (0.59 to 1.39)	0.638
Co-occurrence of unhealthy behaviours										
*- Non-adherence to Mediterranean dietary pattern & insufficient physical activity & smoking*	1.00		1.00		1.00		1.00		1.00	
*- Non-adherence to Mediterranean dietary pattern & insufficient physical activity*			1.08 (0.85 to 1.37)	0.543	1.28 (1.00 to 1.64)	0.049	0.82 (0.62 to 1.09)	0.181	0.61 (0.38 to 0.98)	0.040
*- Non-adherence to Mediterranean dietary pattern & smoking*	1.01 (0.65 to 1.56)	0.962			1.33 (0.90 to 1.94)	0.150	0.82 (0.57 to 1.18)	0.292	0.86 (0.49 to 1.49)	0.582
*- Smoking & insufficient physical activity*	0.97 (0.60 to 1.57)	0.902	1.02 (0.67 to 1.57)	0.912			1.01 (0.69 to 1.49)	0.946	0.89 (0.50 to 1.61)	0.709
Education level										
*- Secondary or higher education*	1.00		1.00		1.00		1.00		1.00	
*- Primary or lower education*	0.98 (0.68 to 1.41)	0.905	1.12 (0.89 to 1.39)	0.339	0.91 (0.73 to 1.13)	0.390	1.00 (0.82 to 1.20)	0.967	1.02 (0.74 to 1.39)	0.924
Employment situation										
*- Employed*	1.00		1.00		1.00				1.00	
*- Unemployed*	0.53 (0.25 to 1.13)	0.098	1.00 (0.71 to 1.41)	0.981	0.88 (0.60 to 1.30)	0.533	0.85 (0.63 to 1.16)	0.304	0.69 (0.39 to 1.23)	0.210
*- Other situations*	1.02 (0.68 to 1.54)	0.907	1.02 (0.79 to 1.31)	0.900	1.12 (0.85 to 1.46)	0.421	1.06 (0.85 to 1.34)	0.600	1.03 (0.71 to 1.49)	0.888

OR, Odds ratio

*In these models, each state of change was considered as a variable with possible values of 0, 0.33, 0.66 and 1 according to the participant was in this state for none, one, two or all three behaviours

### Implementation outcomes

#### Adoption

The overall adoption rate was 48.7% (251 PHC professionals out of 515), ranging by PHC centre from 21.3 to 83.3%.

#### Appropriateness and acceptability

Table 5 shows response rates and results obtained from the questionnaires administered to PHC professionals and participants. Concerning PHC professionals, both early and final appropriateness were perceived as moderate (mean scores < 7). Professionals perceived lower early appropriateness of the intervention targeting smoking cessation than the interventions targeting other health behaviours. In contrast, no differences were found in the final perceived appropriateness of interventions targeting the three health behaviours. For the three health behaviours targeted, early perceived appropriateness was higher than the final. Perceived early acceptability was high (mean scores > 7), whereas final acceptability was moderate (mean scores < 7). Results related to acceptability were comparable with appropriateness, but in this case, final acceptability was lowest for the smoking cessation intervention. Concerning the results obtained in the participants, acceptability was high (mean scores > 8), with no differences observed between initial and final perception. Perceived appropriateness was moderate, with the initial perception worse than the final.

**Table 5:** Description of early and final appropriateness and acceptability of the EIRA intervention as perceived by Primary Health Care professionals and participants. Values are mean (standard deviation). *Minimum score, 0; maximum score, 10*

	Target behaviours			
PHC professionals *Response rates* Early 64.5% (162/251) Final 27.9% (70/251)	Smoking	Insufficient physical activity	Non-adherence to Mediterranean dietary pattern	***P value***†
**Appropriateness (3 items)** ***n*** **= 162**				
Early	6.5 (1.76)	6.7 (1.77)	6.8 (1.79)	0.003
Final	5.8 (2.03)	6.1 (2.01)	6.2 (2.17)	0.099
P value*	0.009	0.024	0.030	
**Acceptability (5 items)** ***n*** **= 70**				
Early	7.3 (1.50)	7.5 (1.26)	7.6 (1.29)	< 0.001
Final	5.8 (1.83)	6.3 (1.67)	6.3 (1.76)	0.010
P value*	< 0.001	< 0.001	< 0.001	
**Participants** *Response rates* Early 17.9% (237/1325) Final 59.5% (788/1325)				
**Appropriateness (5 items)** ***n*** **= 237**				
Early		6.9 (1.41)		
Final		7.2 (1.82)		
P value*		0.020		
**Acceptability (2 items)** ***n*** **= 788**				
Early		8.1 (0.97)		
Final		8.2 (1.41)		
P value*		0.307		

PHC, Primary Health Care

*Student’s t-test

†ANOVA

#### Feasibility

Consent rate was 82.0% (range by PHC centre 43.5–100.0%) and recruitment rate was 65.5% (range 36.2–98.6%). To recruit 1481 participants, 2262 people were invited to participate, a ratio of two offers per one recruit. Intervention uptake was 89.5% (range 76.8–100.0%) and completion rate 74.7% (range 56.5–91.4%).

#### Fidelity of the planned intervention

The individual approach to promote physical activity BC had the highest fidelity (52.2%) and the community approach the lowest (19.1%) (see Table 6). The total percentage of all approaches with fidelity ≥50% was 16.7%. This value ranged from 0 in two centres (H and M) to 83.3 in centre G.

**Table 6:** Fidelity outcomes (%) and Consolidated Framework for Implementation Research (CFIR) construct ratings by PHC centre. Ratings reflects its valence (+ or -) and its strength (weak (1) or strong (2)) of impact on implementation

**PHC centre**	**A**	**B**	**C**	**D**	**E**	**F**	**G**	**H**	**I**	**J**	**K**	**L**	**M**	**Global**			
Individual approach: smoking cessation	63.6	46.8	50.5	35.9	20.6		63.4	26.6	69.4	78.4	43.6	55.2	37.7	48.9			
Individual approach: physical activity	75.7	64.5	54.0	49.0	19.5		68.5	22.4	70.4	63.2	65.5	44.3	41.3	52.2			
Individual approach: Mediterranean dietary pattern	72.0	54.5	35.0	46.2	19.5		53.5	17.2	50.9	48.2	52.3	51.9	33.3	43.5			
Group approach	37.6	63.5	35.6	57.5	58.0		51.4	31.4	44.0	45.4	44.0	59.7	30.3	45.7			
Community approach	12.0	19.1	7.8	5.7	51.1		19.4	21.4	30.6	19.8	2.7	9.9	10.9	19.1			
Group or community approaches	43.6	65.7	37.8	57.3	66.3		57.8	33.9	52.0	48.0	39.5	61.3	34.1	49.7			
% of approaches with fidelity ≥50%	50.0	66.7	33.3	33.3	33.3		83.3	0.0	66.7	33.3	33.3	66.7	0.0	16.7			
**DOMAIN & CONSTRUCTS**														**No. of occurrences**	**Influence of ratings, n (%)**		
															**Positive**	**Negative**	**Neutral or mixed**
**Intervention characteristics**																	
*Intervention Source*†	I	M	x	M	x		I	M	M	M	I	I	I	5	5 (100)	0 (0)	0 (0)
*Adaptability*†	+1	0	x	-1	x		+1	+1	0	+1	0	+2	+1	10	6 (60)	1 (10)	3 (30)
*Complexity*‡	M	-1	x	M	x		M	M	-2	M	0	-1	M	4	0 (0)	3 (75)	1 (25)
**Outer setting**																	
Needs & Resources of Those Served by the Organization*	-1	0	x	M	x		-1	+1	+1	M	-1	0	M	7	2 (29)	3 (42)	2 (29)
*External Policy& Incentives*‡	-2	-2	x	M	x		M	-2	M	0	-2	0	-2	7	0 (0)	5 (71)	2 (29)
**Inner setting**																	
*Structural Characteristics*‡	M	-2	x	M	x		M	-2	M	0	-1	M	-2	5	0 (0)	4 (80)	1 (20)
Culture*	M	-1	x	M	x		+1	-1	+1	M	+1	M	M	5	3 (60)	2 (40)	0 (0)
Relative Priority**	+1	+1	x	M	x		M	-1	+1	-1	M	0	M	6	3 (50)	2 (33)	1 (17)
Leadership Engagement**	M	-2	x	-1	x		M	M	M	0	M	M	M	3	0 (0)	2 (67)	1 (33)
Available Resources**	-2	-2	x	M	x		+1	-2	-1	-1	-2	-2	-2	9	1 (11)	8 (89)	0 (0)
Access to Knowledge & Information**	+1	-1	x	-1	x		-1	-2	0	0	M	-1	-1	9	1 (11)	6 (67)	2 (22)
Formally Appointed Internal Implementation Leaders*	+1	0	x	+1	x		M	-1	M	M	-1	M	-1	6	2 (33)	3 (50)	1 (17)
*External Change Agents*†	+1	0	x	+1	x		M	+1	+1	M	+1	M	+1	7	6 (86)	0 (0)	1 (14)
Reflecting & Evaluating*	+1	M	x	0	x		+1	+1	+2	-1	+1	M	M	7	5 (72)	1 (14)	1 (14)

M denotes that the construct did not emerge; 0 denotes mixed or neutral data; NA, not applicable; I, intervention was developed internally

X denotes that the evaluation was not carried out; The F PHC centre dropped out in preimplementation stage

**denotes a strongly distinguishing construct; *denotes a weakly distinguishing construct, † denotes not distinguishing construct but with positive influence, ‡ denotes not distinguishing construct but with negative influence

### Determinants of implementation effectiveness/success

A total of 27 CFIR constructs emerged in the focus groups, and no new code was identified from the data. Eight constructs distinguished between high and low implementation. Six other constructs were not distinguishing, but they positively or negatively influenced the implementation in most PHC centres. Table 6 shows these 14 constructs and their ratings for each PHC centre. Details of the most salient constructs are described below. Table 7 shows representative quotes from PHC professionals for all these constructs. For the other CFIR constructs, no sufficient data emerged to assess their influence on implementation. Supplementary file 3 provides detailed descriptions of ratings and correlations of the 27 constructs, with intervention fidelity for each approach.

**Table 7:** Representative quotes on the implementation of the EIRA intervention according to their negative or positive influence

CFIR Construct/Domain (Definition)	Positive influence	Negative influence
**Intervention source/ Intervention characteristics** (Perception of key stakeholders about whether the intervention is externally or internally developed)	“Well, the intervention has been designed by the people that work in these issues, people in primary care who I believe are working more on these issues” (PHC centre M)	
**Adaptability/Intervention characteristics** (The degree to which an intervention can be adapted, tailored, refined, or reinvented to meet local needs)	“Because there was the flexible part where you conduct the visit like, a bit following the needs of the patients …” (PHC centre J) “There was no strict protocol saying you have to give the form, you must do this, so naturally, we have adapted it to our own practice, because there was no specific rule on how to do it. I think we had this freedom and we have delivered.” (PHC centre G)	I feel it was something recycled, that was already there, and when people read it’s just another leaflet, if it focused more on our customs or our ways, maybe people would pay more attention (PHC centre D)
**Complexity/ Intervention characteristics** (Perceived difficulty of the intervention, reflected by duration, scope, radicalness, disruptiveness, centrality, and intricacy and number of steps required to implement)		“If they had 3 problems (behaviours), for instance, better stepwise, not everything in the same session … For me it’s better stepwise. Maybe 2, but 3 is complicated. If you are going to introduce changes in food, I think it’s too much change … “(PHC centre B)
**Needs & Resources of Those Served by the Organization/Outer setting (**The extent to which patient needs, as well as barriers and facilitators to meet those needs, are accurately known and prioritized by the organization)	I think for many patients this research has been a push, they had set aside these things and now they thought “this is the time!”. An opportunity for them, and many are really thankful. Because of personal reasons, for some it has not been very successful, for others it has been very beneficial, and they are very thankful, and they explain this to me when I see them again, some feel really happy about it. (PHC centre I)	“I think people don’t want to take responsibility for anything. And parents arrive to the emergency room and say “I believe they have a fever”. They have not even checked with the thermometer! They don’t want responsibilities. People don’t want, and if you don’t take responsibility, how can you change attitude? “(PHC centre A) Yes, because they thought we would provide a miracle diet just for them, and it was of course impossible, what we did was general, explaining types of diet, how to shop for food … For me, when we prepared it I found it very practical, but of course, many came with “but my problem is, my problem is”, “for this specific thing you have to see your EIRA nurse to get this, this is a bit more general”, we explained what to look for in the labels, the calories, saturated fat and such, but naturally they expected a form with the miracle. That was not happening, what can you do. (PHC centre G) “With respect to physical activity, the resources of the Community were limited.” “There were no resources.” “There were none. Only the Red Cross for people over 65 years and for something free of charge, not for profit, we had little. Neighbourhood associations …” (PHC centre K)
**External Policy &Incentives/Outer setting** (A broad construct that includes external strategies to spread interventions, including policy and regulations (governmental or other central entity), external mandates, recommendations and guidelines, pay-for-performance, organization, and public or benchmark reporting)		“… adapting to a different working method seems easy but it’s not, because you have to break habits that are difficult to change, particularly from a certain age, where you have another perspective of things” (PHC centre M) “… at least most nursing staff comes from workplaces very different from primary care, they come at an age when it’s very difficult that the years or the few years until retirement they change” (PHC centre M) “I think, perhaps, we are not supported by the management, the preventive policy …”; “This requires institutional policies from the start. But this has not happened yet” (PHC centre B)
**Structural characteristics/Inner setting** (The social architecture, age, maturity, and size of an organization)	I believe that having a small team has helped (PHC centre J)	The volatility of staff. You have a team and suddenly half of them are not there anymore. (PHC centre B)
**Culture/Inner setting** (Norms, values, and basic assumptions of a given organisation)	“I believe this is not your average centre, above the area, […] I believe that yes, always … in trying new things” (PHC centre K)	The outlook needs to change [..] for managers, for professionals, … (PHC centre B)
**Relative priority/Inner setting** (Individuals’ shared perception of the importance of the implementation within the organization)	“… losing weight is more important than the pharyngitis” (PHC centre “A”) “I believe that a GP or a primary care nurse should work on this half of their time” (PHC centre “A”)	“There have been periods with a full agenda … And other issues have been prioritized.” (PHC centre “J”) “I don’t think a project about this is a priority” (PHC centre H)
**Leadership engagement/Inner setting** (Commitment, involvement, and accountability of leaders and managers with the implementation)		The only preventive activity that I know the director is involved in are vaccinations. And this for me is the example, the administration needs to organise, organise and offer the means to implement. (PHC centre B) I think that they should be involved, you know, what we discussed, that to achieve something it’s not only at our level, also for the managers (PHC centre B)
**Available resources/Inner setting** (The level of resources dedicated for implementation and on-going operations, including money, training, education, physical space, and time)	For this to be feasible, we already said, we need time, maybe we were already doing this, maybe in other centres because of high demand could not integrate these activities in their daily practice. So this is the time needed (PHC centre G)	“We need more space” (PHC centre K) “We have too many patients, we are too willing, we have too much material. What we lack is time, that’s it, in short.” (PHC centre A)
**Access to Knowledge & Information/Inner setting** (Ease of access to digestible information and knowledge about the intervention and how to incorporate it into work tasks)	And yet, the two PBI sessions (in-person group feedback session), maybe then I don’t know, us discussing, being there even discussing something among us, because at home on your own you go over it time and time again … But when you are with other people and discuss it, maybe others see what you don’t see, or you say “maybe not, I thought it was perfect and the reality is it can improve”. (PHC centre J)	Yes, I mean, there were two trainings. One face to face, which was good because you could interact, yes, and then it’s true that people were rather unhappy with the online interview course, people didn’t like it. We did not like it, it was not useful, … (PHC centre G)
**Formally appointed internal implementation leaders/Process** (Individuals from within the organization who have been formally appointed with responsibility for implementing an intervention as coordinator, project manager, team leader, or other similar role)	“There is always somebody appointed to lead, isn’t it? That’s my experience, anyhow. So that nobody can say …”; (PHC centre A)	In the community commission, where I was, it helped a bit in connecting with other people, I used to call, … and, I don’t remember anybody leading, more as a team. I really don’t remember who the leader was. Well, then, it has worked, but I really don’t think it’s because of the leadership. (PHC centre H)
**External change agents/Process** (Individuals who are affiliated with an outside entity who formally influence or facilitate intervention decisions in a desirable direction)	“thanks to your help, well, we have been able to do it” (PHC centre D)	
**Reflecting & evaluating/Process** (Quantitative and qualitative feedback about the progress and quality of implementation accompanied with regular personal and team debriefing about progress and experience)	And what you used to send, the bulletins, were also very useful. (PHC centre I)	Well, maybe the sessions, when there was a session just for EIRA, we asked “do you have any issue to discuss? No, well...”, then each of us tends to their business (PHC centre J)

*Needs and Resources of Those Served by the Organisation* was a weakly distinguishing construct of fidelity of the individual approach to dietary BC (rho = − 0.866; *P* = 0.058) and the community approach (rho = 0.686; *P* = 0.058). PHC professionals expressed that participants did not feel the need to assume their self-care. They also felt that their expectations were closer to “miracle diets” than following a set of guidelines to make their diet healthier. Some comments pointed to a lack of resources in the community to carry out physical activity programs. Nevertheless, there was also positive feedback on patients’ satisfaction with the intervention. *Culture* weakly distinguished between high and low fidelity of the individual approach to promote physical activity BC (rho = 0.866; *P* = 0.058). Positive statements highlighted the culture of continuous innovation at the PHC centre, while negative statements indicated a reluctance to change and implement new practices. *Relative priority* was identified as a distinguishing construct with high and low intervention fidelity, strong for the individual approach in promoting physical activity BC (rho = 0.833; *P* = 0.039) and weak in promoting dietary BC (rho = 0.802; *P* = 0.055). This construct emerged in six PHC centres. Some professionals argued that the EIRA intervention was considered a low priority compared to more immediate activities such as acute health care. In contrast, professionals from PHC centres where the intervention fidelity was greater (for example, PHC centre A) stated that they believed it was crucial to implement this kind of interventions in PHC. *Leadership engagement* was strongly inversely correlated with the intervention fidelity of the group approach (rho = − 1.000; *P* = 0.010). This construct only emerged in three PHC centres. The influence was negative in two PHC centres, but the centre with the best intervention fidelity showed the strongest negative influence of the construct. The professionals of these PHC centres stated that the managers’ commitment, participation, and accountability did not facilitate the intervention. *Available resources* strongly distinguished between low and high fidelity of the individual approach to promote smoking cessation (rho = 0.667; *P* = 0.050) and weakly in the community approach (rho = 0.588; *P* = 0.096). This construct had a weak positive influence in only one centre (PHC centre G); in the remaining PHC centres, the influence was negative. Most comments from professionals were related to the lack of time and physical space to carry out the intervention. Positive comments reported that this kind of activities had already been integrated and that the PHC centre already allocated resources to do so, especially time. *Access to Knowledge & Information* was correlated with intervention fidelity, strongly in individual approaches to promote smoking cessation (rho = 0.853; *P* = 0.003) and physical activity BC (rho = 0.780; *P* = 0.013). This construct emerged in nine PHC centres, and it had a weak positive influence only in one centre (PHC centre A). The PHC centre with the lowest intervention fidelity was the centre where the most strongly negative influence (PHC centre H). Most professionals emphasized the lack of practicality and the challenges encountered about the online training in motivational interviewing. However, the in-person group feedback sessions were positively evaluated. *Formally appointed internal implementation leaders* weakly distinguished between low and high fidelity of group or community approaches (rho = 0.741; *P* = 0.092). It emerged in six PHC centres, and the three centres with the lowest fidelity percentages in these approaches had a weak negative influence. The negative comments mainly pointed at the lack of an internal leader to implement the EIRA intervention. *Reflecting and Evaluating* weakly distinguished between high and low fidelity of the individual approach to promote physical activity BC (rho = 0.759; *P* = 0.080). Only one PHC centre showed a weak negative influence of this construct. Professionals reported that they had not collectively and systematically addressed the development of the implementation of the intervention. They also highlighted the usefulness of newsletters reporting on the development of the intervention.

Three non-distinguishing constructs showed an overall positive influence: *Intervention source, Adaptability* and *External Change Agents*. The EIRA intervention was perceived as internally developed, and PHC professionals acknowledged that PHC researchers had developed the intervention. Most PHC professionals considered that the EIRA intervention was adaptable. PHC professionals highlighted the advantages of a non-rigid protocol that could be adapted to the context and needs of participants. However, there were some negative statements regarding the complex adaptation of the Mediterranean dietary pattern in cultural contexts where another dietary pattern predominated. PHC professionals emphasized the engagement of the facilitators (members of the research team) and their role in implementing the intervention.

The remaining three non-distinguishing constructs showed an overall negative influence: *Complexity*, *External Policy & Incentives* and *Structural Characteristics*. PHC professionals highlighted the difficulty of simultaneously approaching two or three health behaviours and conveyed a preference for tackling BC individually. Some comments related to job allocation policies, particularly regarding nurses with a long history of hospital care applying for PHC jobs without prior training for this healthcare setting. Lack of alignment of the intervention with the organisation’s objectives was identified as a barrier. PHC professionals noted that their organisation prioritised curative versus preventive and health promotion care. Most statements mentioned a lack of continuity of the workforce. One of the PHC centres attributed a positive value to the small size of the PHC team, which facilitated the implementation of the intervention.

## Discussion

The EIRA study was designed to determine the effectiveness of a MHBC primary care intervention and its implementation strategy. Results indicate that the intervention was more effective and had a higher impact in promoting MHBC than usual care at 12-month follow-up in people aged 45 to 75. Among the three target behaviours, the intervention was more effective in promoting dietary BC. About implementation outcomes, adoption was moderate and wide variability was observed between PHC centres. The EIRA intervention was perceived as appropriate and acceptable by PHC professionals and participants. The intervention was feasible, the required number of participants was recruited, and the percentage of losses was lower than anticipated. However, the fidelity level of the planned intervention was low, with only the individual approach to promote physical activity BC exceeding 50%.

The EIRA study has several strengths. We intended to develop a flexible intervention that could be adapted to different PHC settings, and the intervention’s design was based on the results of previous phases of the study. Additionally, we used theoretical frameworks for the study design, data collection, analysis, interpretation, and evaluation. CFIR constructs were used as variables in investigating implementation outcomes, and we have been able to provide explicit links between them to help better understand intervention effects and implementation barriers. However, several limitations exist. Baseline imbalance and confounding bias can occur in cluster randomised trials [33]. There were a higher proportion of participants in pre-action stages in the control group than in the intervention group, especially in the preparation stage. Since this imbalance might have contributed to overestimating the effect of the intervention, we have included the baseline stages of change in the logistic regression models as covariates. However, there may be unknown or unmeasured confounders for which statistical analysis has been unable to adjust. To avoid this bias, we could have used an independent recruiter blinded to allocation. However, this hybrid trial is inherently pragmatic since it was designed to evaluate the feasibility of recruitment in the real practice of PHC professionals. Furthermore, we have assessed the fidelity outcome using an indirect measure based on adherence to activities instead of on the professional’s skills to carry them out, which might underrepresent true fidelity. However, the differential association with the CFIR constructs supported its discriminant validity.

In accordance with the current results, previous studies have demonstrated that MHBC interventions can successfully promote small improvements in dietary and physical activity behaviours and smoking cessation [6]. The dietary BC was more relevant and significant, in agreement with other studies on MHBC interventions in PHC settings and population-based studies [55, 56]. Although different studies show that MHBC interventions can slightly increase physical activity, the EIRA intervention did not make statistically significant BC in physical activity [6]. Fernald et al. [55] found that the MHBC intervention was only effective in promoting physical activity BC in two of the seven participating networks, and Campbell et al. [56] did not observe any effect of the intervention on this behaviour. In our study, the individual approach to promote physical activity BC had the highest fidelity (52.2%). However, of all individual approaches, physical activity was particularly complemented by the community approach, which presented the lowest fidelity (19.1%). We believe that this might account for the low effectiveness of the intervention in changing this behaviour. No effect was observed regarding smoking cessation. Since changes in smoking are negatively associated with changes in other behaviours, an individual health BC intervention might be more adequate for smoking cessation [6].

Several factors have influenced the effectiveness of the EIRA intervention compared to usual care. The training centre status had a negative effect on physical BC. The study by March et al. [10] found a negative association between PHC training centres and health-promoting community activities. Recently, PHC interventions to increase physical activity have been integrated into usual practice in Spain [41, 57], and non-training PHC centres might have been more motivated to promote physical activity BC. Additionally, the preparation stage appears to promote change for any of the three behaviours studied. Previous research shows that people in a higher stage for one behaviour are also more likely to be in a higher stage for another behaviour [58]. Co-occurrence of non-adherence to a Mediterranean dietary pattern and insufficient physical activity explained dietary BC more than any other combination of concurrent behaviours. In line with other studies [4], it was the most prevalent health behaviour combination (56.4% of the study population), which may have increased its explanatory potential over the other three behaviour combinations. Dietary and physical activity behaviours are positively correlated, and while it has been suggested that change in one behaviour facilitates change in the other [59, 60], in our study, the concurrence of these unhealthy behaviours was negatively related to MHBC. These results are consistent with further MHBC research, which found that of the two behaviours, only the diet improved [56]. These inconsistencies highlight the need to generate more evidence on predictors of coaction in MHBC interventions [61, 62].

Very little was found in the literature on the implementation of MHBC interventions in PHC. The results of the implementation of the EIRA study agree with the results observed by Martinez et al. [63], stating that *Intervention Source* and *Reflecting and Evaluating* are CFIR constructs related to implementation success. Similarly, both studies found that some constructs such as *Complexity*, *External Policy and Incentives*, and *Structural characteristics* were not associated with implementation success, perhaps because they negatively influenced all PHC centres. In this study, the influence of some of these constructs even determined the dropout of a PHC centre. However, the current study’s findings do not support that *Adaptability* does not positively influence any PHC centres. On the other hand, we found several determinants associated with implementation success such as *Relative Priority, Available Resources, Leadership engagement,* and *Culture* that Martinez et al. [63] did not identify. *Available resources* are a commonly perceived barrier by PHC professionals to the integration of health promotion activities into daily practice [11, 63]. *Leadership Engagement* was negatively correlated with the intervention fidelity of the group approach, i.e., less *Leadership Engagement* more intervention fidelity, probably because the predominance of a laissez-faire leadership style leaves a significant degree of participation and responsibility in organisational decision-making to professionals [64]. Leadership profoundly influences any organisation’s culture [65], which might explain why the *Culture* construct also emerged as a distinguishing construct.

The findings of this study agree with the approach proposed by Prochaska [62] and highlight implications for the integration of MHBC interventions in PHC settings, and they can help to reach a greater understanding of how MHBC interventions work in PHC settings. Furthermore, healthcare services are currently facing the difficult task of providing care due to limited resources and unlimited demands, so priority setting and rationing are applied. EIRA study shows that there are factors in Spanish PHC closely related to these actions that have hampered the MHBC intervention. For MHBC interventions to succeed, their relative priority over other interventions needs to increase. However, the organisational culture of current PHC services remains largely disease-oriented rather than person-centred [66]. The readiness of PHC services for the implementation of MHBC interventions requires resources and leadership. Evidence points to the strong relationship between the adequacy of an organisation’s resources and the adoption of innovative practices [67]. In addition, facilitative leadership is essential to shift organisations toward a culture of innovation [68]. Significantly, PHC professionals considered the implementation of the intervention complex, mainly because they had to tackle three unhealthy behaviours at the same time. Unlike interventions aimed at changing a single behaviour, the field of MBHC is still relatively unknown. Therefore, it is essential to increase efforts to improve the knowledge and dissemination of MHBC interventions [61].

Further studies should elucidate the factors influencing MHBC, such as life skills and social, cultural or environmental factors, in order to generate evidence to create and invest in resources that can modify them [61]. Coaction is also an aspect of MHBC on which research is needed. It occurs when “taking effective action on one behaviour increases the odds of taking effective action on a second behaviour” and reflects behaviours that change together and increases the impact of MHBC interventions [62]. Finally, incorporating MHBC interventions in PHC settings is a significant challenge. Implementation research can advance understanding of factors that facilitate or hinder the implementation of MHBC interventions and provide evidence about the effectiveness of different implementation strategies [69].

## Conclusions

In summary, progress in the MHBC field could improve the prevention and management of NCDs, and PHC can play an essential role in achieving it. However, research conducted on MHBC interventions and their implementation in PHC settings remains insufficient. EIRA study provides evidence about the effectiveness of an intervention to promote MHBC and its implementation in PHC setting. Results of the study will increase the knowledge about which implementation strategies are the most suitable in the context of the PHC, helping a greater integration of MHBC interventions in the everyday practice of the PHC professionals.

## Supplementary Information

**Additional file 1.**

**Additional file 2.**

**Additional file 3.**

## Data Availability

The datasets used and analysed during this study are available from the corresponding author on reasonable request.

## Abbreviations

BC: Behaviour Change
BMI: Body Mass Index
CFI: Comparative Fit Index
CFIR: Consolidated Framework for Implementation Research
CI: Confidence Interval
COPD: Chronic Obstructive Pulmonary Disease
HDL: High-Density Lipoprotein
IQR: Interquartile Range
LDL: Low-Density Lipoprotein
MAR: Missing At Random
MHBC: Multiple Health Behaviour Change
MRC: Medical Research Council
NCDs: Non-Communicable Diseases
NNC: Number Needed for Change
PHC: Primary Health Care
OR: Odds Ratio
RMSEA: Root Mean Square Error of Approximation
SD: Standard deviation
SMS: Short Message Service
