# Supplementary material for: Multiple health behaviour change primary care intervention for smoking cessation, physical activity and healthy diet in adults 45 to 75 years old (EIRA study): a hybrid effectiveness-implementation cluster randomised trial
Source: BMC Public Health. 2021 Dec 4;21:2208. doi: 10.1186/s12889-021-11982-4 (PMC8642878; doi:10.1186/s12889-021-11982-4)
Supplement: Supplementary file 1 — Additional file 1. [file 12889_2021_11982_MOESM1_ESM.docx]

**Questionnaire (PHC professionals’ version)**

This is a questionnaire of the EIRA project: “Hybrid trial to assess a multirisk complex evaluation to promote healthy behaviour in primary care patients 45-75 years old: EIRA 3 study”.

We want to know your **opinions and expectations** about the intervention **before the study starts**. This information will only be used for research purposes and the data will remain confidential at all times.

Please indicate your agreement with the following statements, taking into account that “0” indicates maximum disagreement and “10” maximum agreement

1. The intervention will help my patients…

|  | Totally disagree | |  |  | Don’t agree nor disagree | | |  |  | Totally agree | |
| --- | --- | --- | --- | --- | --- | --- | --- | --- | --- | --- | --- |
|  |  | |  |  |  | | |  |  |  | |
| ... eat more healthily | 0 | 1 | 2 | 3 | 4 | 5 | 6 | 7 | 8 | 9 | 10 |
|  |  |  |  |  |  |  |  |  |  |  |  |
| … exercise more | 0 | 1 | 2 | 3 | 4 | 5 | 6 | 7 | 8 | 9 | 10 |
|  |  |  |  |  |  |  |  |  |  |  |  |
| ...quit smoking | 0 | 1 | 2 | 3 | 4 | 5 | 6 | 7 | 8 | 9 | 10 |

1. Compared to other interventions that are already incorporated into my clinical practice, this intervention will better help my patients…

|  | Totally disagree | |  |  | Don’t agree nor disagree | | |  |  | Totally agree | |
| --- | --- | --- | --- | --- | --- | --- | --- | --- | --- | --- | --- |
|  |  | |  |  |  | | |  |  |  | |
| ... eat more healthily | 0 | 1 | 2 | 3 | 4 | 5 | 6 | 7 | 8 | 9 | 10 |
|  |  |  |  |  |  |  |  |  |  |  |  |
| … exercise more | 0 | 1 | 2 | 3 | 4 | 5 | 6 | 7 | 8 | 9 | 10 |
|  |  |  |  |  |  |  |  |  |  |  |  |
| ...quit smoking | 0 | 1 | 2 | 3 | 4 | 5 | 6 | 7 | 8 | 9 | 10 |

1. This intervention is important to help my patients …

|  | Totally disagree | |  |  | Don’t agree nor disagree | | |  |  | Totally agree | |
| --- | --- | --- | --- | --- | --- | --- | --- | --- | --- | --- | --- |
|  |  | |  |  |  | | |  |  |  | |
| ... eat more healthily | 0 | 1 | 2 | 3 | 4 | 5 | 6 | 7 | 8 | 9 | 10 |
|  |  |  |  |  |  |  |  |  |  |  |  |
| … exercise more | 0 | 1 | 2 | 3 | 4 | 5 | 6 | 7 | 8 | 9 | 10 |
|  |  |  |  |  |  |  |  |  |  |  |  |
| ...quit smoking | 0 | 1 | 2 | 3 | 4 | 5 | 6 | 7 | 8 | 9 | 10 |

1. This intervention will be useful to help my patients…

|  | Totally disagree | |  |  | Don’t agree nor disagree | | |  |  | Totally agree | |
| --- | --- | --- | --- | --- | --- | --- | --- | --- | --- | --- | --- |
|  |  | |  |  |  | | |  |  |  | |
| ... eat more healthily | 0 | 1 | 2 | 3 | 4 | 5 | 6 | 7 | 8 | 9 | 10 |
|  |  |  |  |  |  |  |  |  |  |  |  |
| … exercise more | 0 | 1 | 2 | 3 | 4 | 5 | 6 | 7 | 8 | 9 | 10 |
|  |  |  |  |  |  |  |  |  |  |  |  |
| ...quit smoking | 0 | 1 | 2 | 3 | 4 | 5 | 6 | 7 | 8 | 9 | 10 |

1. It will be easy to integrate into my daily practice the intervention aimed at …

|  | Totally disagree | |  |  | Don’t agree nor disagree | | |  |  | Totally agree | |
| --- | --- | --- | --- | --- | --- | --- | --- | --- | --- | --- | --- |
|  |  | |  |  |  | | |  |  |  | |
| ... promoting healthy eating behaviours | 0 | 1 | 2 | 3 | 4 | 5 | 6 | 7 | 8 | 9 | 10 |
|  |  |  |  |  |  |  |  |  |  |  |  |
| … promoting physical activity | 0 | 1 | 2 | 3 | 4 | 5 | 6 | 7 | 8 | 9 | 10 |
|  |  |  |  |  |  |  |  |  |  |  |  |
| ...reducing smoking | 0 | 1 | 2 | 3 | 4 | 5 | 6 | 7 | 8 | 9 | 10 |

1. With the resources I have, it will be easy to implement the intervention aimed at …

|  | Totally disagree | |  |  | Don’t agree nor disagree | | |  |  | Totally agree | |
| --- | --- | --- | --- | --- | --- | --- | --- | --- | --- | --- | --- |
|  |  | |  |  |  | | |  |  |  | |
| ... promoting healthy eating behaviours | 0 | 1 | 2 | 3 | 4 | 5 | 6 | 7 | 8 | 9 | 10 |
|  |  |  |  |  |  |  |  |  |  |  |  |
| … promoting physical activity | 0 | 1 | 2 | 3 | 4 | 5 | 6 | 7 | 8 | 9 | 10 |
|  |  |  |  |  |  |  |  |  |  |  |  |
| ...reducing smoking | 0 | 1 | 2 | 3 | 4 | 5 | 6 | 7 | 8 | 9 | 10 |

1. Considering the characteristics and the context of my patients, it will be easy to adapt the intervention aimed at...

|  | Totally disagree | |  |  | Don’t agree nor disagree | | |  |  | Totally agree | |
| --- | --- | --- | --- | --- | --- | --- | --- | --- | --- | --- | --- |
|  |  | |  |  |  | | |  |  |  | |
| ... promoting healthy eating behaviours | 0 | 1 | 2 | 3 | 4 | 5 | 6 | 7 | 8 | 9 | 10 |
|  |  |  |  |  |  |  |  |  |  |  |  |
| … promoting physical activity | 0 | 1 | 2 | 3 | 4 | 5 | 6 | 7 | 8 | 9 | 10 |
|  |  |  |  |  |  |  |  |  |  |  |  |
| ...reducing smoking | 0 | 1 | 2 | 3 | 4 | 5 | 6 | 7 | 8 | 9 | 10 |

1. Compared to similar interventions, I will need more time for the intervention aimed at…

|  | Totally disagree | |  |  | Don’t agree nor disagree | | |  |  | Totally agree | |
| --- | --- | --- | --- | --- | --- | --- | --- | --- | --- | --- | --- |
|  |  | |  |  |  | | |  |  |  | |
| ... promoting healthy eating behaviours | 0 | 1 | 2 | 3 | 4 | 5 | 6 | 7 | 8 | 9 | 10 |
|  |  |  |  |  |  |  |  |  |  |  |  |
| … promoting physical activity | 0 | 1 | 2 | 3 | 4 | 5 | 6 | 7 | 8 | 9 | 10 |
|  |  |  |  |  |  |  |  |  |  |  |  |
| ...reducing smoking | 0 | 1 | 2 | 3 | 4 | 5 | 6 | 7 | 8 | 9 | 10 |

1. It will be worth investing more time in the intervention aimed at …

|  | Totally disagree | |  |  | Don’t agree nor disagree | | |  |  | Totally agree | |
| --- | --- | --- | --- | --- | --- | --- | --- | --- | --- | --- | --- |
|  |  | |  |  |  | | |  |  |  | |
| ... promoting healthy eating behaviours | 0 | 1 | 2 | 3 | 4 | 5 | 6 | 7 | 8 | 9 | 10 |
|  |  |  |  |  |  |  |  |  |  |  |  |
| … promoting physical activity | 0 | 1 | 2 | 3 | 4 | 5 | 6 | 7 | 8 | 9 | 10 |
|  |  |  |  |  |  |  |  |  |  |  |  |
| ...reducing smoking | 0 | 1 | 2 | 3 | 4 | 5 | 6 | 7 | 8 | 9 | 10 |

Please add any comments and suggestions in this blank space:

………………………………………………………………………………………………………………………………………………………………………………………………………………………………………………………………………………………………………………………………………………………………………………………………………………………………………………………………………………………………………………………………………………………………………………………………………………………………………………………………………………………………………………………………………………………………………………………………………………………………………………………………………………………………………………………………………………………………………………………………………………………………………………………………………………………………

**Thanks for your time**

**Questionnaire (participants’ version)**

This is a questionnaire of the study in which you participate. We want to know what you think about some aspects of this research. Your opinions will be used to provide more tailored health services**. The analysis of these data is confidential.**

We would like to know your opinion on the advice (suggestions, clarifications…) provided during this visit. Please indicate your agreement with the following statements, taking into account that “0” indicates maximum disagreement and “10” maximum agreement.

.

1. **At this point, how feasible do you consider the advice (suggestions, clarifications…) you have received to improve your health?**

| 0 | 1 | 2 | 3 | 4 | 5 | 6 | 7 | 8 | 9 | 10 |
| --- | --- | --- | --- | --- | --- | --- | --- | --- | --- | --- |
| Not feasible at all | | |  | Somewhat feasible | | |  | Very feasible | | |

1. **At this point, how successful do you think the advice (suggestions, clarifications…) received to reduce the risks to your health will be?**

| 0 | 1 | 2 | 3 | 4 | 5 | 6 | 7 | 8 | 9 | 10 |
| --- | --- | --- | --- | --- | --- | --- | --- | --- | --- | --- |
| No successful at all | | |  | Somewhat successful | | |  | Very successful | | |

1. **Would you recommend to a friend with similar health problems the advice (suggestions, clarifications…) received to improve your health?**

| 0 | 1 | 2 | 3 | 4 | 5 | 6 | 7 | 8 | 9 | 10 |
| --- | --- | --- | --- | --- | --- | --- | --- | --- | --- | --- |
| Not at all | | |  | Maybe | | |  | Very much | | |

1. **Could you clearly understand the advice (suggestions, clarifications…) provided by the doctor/nurse?**

| 0 | 1 | 2 | 3 | 4 | 5 | 6 | 7 | 8 | 9 | 10 |
| --- | --- | --- | --- | --- | --- | --- | --- | --- | --- | --- |
| Not at all | | |  | Somewhat | | |  | Very much | | |

1. **How easy will it be to implement the advice (suggestions, clarifications…) provided with your resources?**

| 0 | 1 | 2 | 3 | 4 | 5 | 6 | 7 | 8 | 9 | 10 |
| --- | --- | --- | --- | --- | --- | --- | --- | --- | --- | --- |
| Not easy at all to implement | | |  | Somewhat easy to implement | | |  | Very easy to implement | | |

1. **Do you think that the advice (suggestions, clarifications…) provided adapt to your working, family and social context?**

| 0 | 1 | 2 | 3 | 4 | 5 | 6 | 7 | 8 | 9 | 10 |
| --- | --- | --- | --- | --- | --- | --- | --- | --- | --- | --- |
| Not at all | | |  | Somewhat | | |  | Very much | | |

1. **Do you think that the advice (suggestions, clarifications…) provided is useful to improve your health?**

| 0 | 1 | 2 | 3 | 4 | 5 | 6 | 7 | 8 | 9 | 10 |
| --- | --- | --- | --- | --- | --- | --- | --- | --- | --- | --- |
| Not useful at all | | |  | Somewhat useful | | |  | Very useful | | |

Please add any comments and suggestions in this blank space:

………………………………………………………………………………………………………………………………………………

………………………………………………………………………………………………………………………………………………

………………………………………………………………………………………………………………………………………………

………………………………………………………………………………………………………………………………………………

**Remember to return this questionnaire to your health centre. Many thanks for your cooperation.**
