# Supplementary material for: Multiple health behaviour change primary care intervention for smoking cessation, physical activity and healthy diet in adults 45 to 75 years old (EIRA study): a hybrid effectiveness-implementation cluster randomised trial
Source: BMC Public Health. 2021 Dec 4;21:2208. doi: 10.1186/s12889-021-11982-4 (PMC8642878; doi:10.1186/s12889-021-11982-4)
Supplement: Supplementary file 2 — Additional file 2. [file 12889_2021_11982_MOESM2_ESM.docx]

**Examples of the questions used in the interview guide**

**Intervention Characteristics**

**Intervention Source**

1. Who developed the intervention?
   - What is your opinion of this group/individual?
2. *This question is also related to Process: Engaging. Code responses related to source of the intervention here. Code responses related to who participated in the decision process under Engaging, as an indication of early (or not) engagement. Participation in decision-making is an effective engagement strategy to help people feel ownership of the intervention.*

Why is the intervention being implemented in your setting?

- - Who decided to implement the intervention?
  - How was the decision made to implement the intervention?

**Evidence Strength & Quality**

1. What kind of information or evidence are you aware of that shows whether or not the intervention will work in your setting?
   - What evidence have you heard about from your own research? Practice guidelines? Published literature? Co-workers? Other settings?
   - How does this knowledge affect your perception of the intervention?
2. *In a healthcare setting, influential stakeholders may include influential and well-respected clinicians, where as in an education setting, this may include influential and well-respected teachers or educators.*

What do influential stakeholders think of the intervention?

- - What do administrative or other leaders think of the intervention?

1. What kind of supporting evidence or proof is needed about the effectiveness of the intervention to get staff on board?
   - Co-workers? Administrative leaders?

**Relative Advantage**

*Coding between Tension for Change, Relative Advantage, and Needs and Resources of Those Served by the Organization will be nuanced but here are some general guidelines:*

- *Tension for Change:
  Statements that demonstrate a strong need for the intervention and/or that the current situation is untenable.*
- *Relative Advantage:
  Statements that demonstrate the intervention is better (or worse) than existing programs.*
- *Needs and Resources:
  Statements regarding specific needs of individuals that demonstrate a need for the intervention, but do not necessarily represent a strong need or an untenable status quo.*

1. How does the intervention compare to other similar existing programs in your setting?
   - What advantages does the intervention have compared to existing programs?
   - What disadvantages does the intervention have compared to existing programs?
2. How does the intervention compare to other alternatives that may have been considered or that you know about?
   - What advantages does the intervention have compared to these other programs?
   - What disadvantages does the intervention have compared to these other programs?
3. Is there another intervention that people would rather implement?
   - Can you describe that intervention?
   - Why would people prefer the alternative?

**Adaptability**

1. What kinds of changes or alterations do you think you will need to make to the intervention so it will work effectively in your setting?
   - Do you think you will be able to make these changes? Why or why not?
2. Who will decide (or what is the process for deciding) whether changes are needed to the intervention so that it works well in your setting?
   - How will you know if it is appropriate to make any changes?
3. Are there components that should not be altered?
   - Which ones should not be altered?

**Trialability**

1. Will the intervention be piloted prior to full-scale implementation?
   - [If Yes] Can you describe what your plans are for piloting the intervention?
   - [If Yes] What will the pilot look like?
2. Do you think it would be possible to pilot the intervention before making it available to everyone?
   - Why or why not?
   - Would this be helpful?

**Complexity**

- *This construct addresses the complexity of the intervention, not the complexity of the implementation. Challenges related to implementation should be coded to the appropriate CFIR code, e.g. challenges with engaging staff should be coded to Engaging: Key Stakeholders or challenges related to making the intervention a priority for leadership should be (double) coded to Leadership Engagement and Relative Priority.*

1. How complicated is the intervention?
   - Please consider the following aspects of the intervention: duration, scope, intricacy and number of steps involved and whether the intervention reflects a clear departure from previous practices.

**Design Quality & Packaging**

1. What is your perception of the quality of the supporting materials, packaging, and bundling of the intervention for implementation?
   - Why?
2. What supports, such as online resources, marketing materials, or a toolkit, are available to help you implement and use the intervention?
   - How do you access these materials?
3. How will available materials affect implementation in your setting?
