# Supplementary material for: Multiple health behaviour change primary care intervention for smoking cessation, physical activity and healthy diet in adults 45 to 75 years old (EIRA study): a hybrid effectiveness-implementation cluster randomised trial
Source: BMC Public Health. 2021 Dec 4;21:2208. doi: 10.1186/s12889-021-11982-4 (PMC8642878; doi:10.1186/s12889-021-11982-4)
Supplement: Supplementary file 3 — Additional file 3. [file 12889_2021_11982_MOESM3_ESM.docx]

**Constructs identified by PHC professionals associated to the fidelity to the intervention**

|  |  | **Individual approaches** | | |  |  |  |
| --- | --- | --- | --- | --- | --- | --- | --- |
|  | **Number of times the construct emerged** | **Smoking cessation** | **Physical activity behaviour change** | **Dietary behaviour change** | **Group approach** | **Community approach** | **Group or community approach** |
| **OUTER SETTING** |  |  |  |  |  |  |  |
| Needs & Resources of Those Served by the Organization | **7** |  |  | ***** |  | ***** |  |
| **INNER SETTING** |  |  |  |  |  |  |  |
| Culture | **5** |  | ***** |  |  |  |  |
| Relative Priority | **6** |  | ****** | ***** |  |  |  |
| Leadership Engagement | **3** |  |  |  | ****** |  |  |
| Available resources | **9** | ****** |  |  |  | ***** |  |
| Access to Knowledge & Information | **9** | ****** | ****** |  |  |  |  |
| **PROCESS** |  |  |  |  |  |  |  |
| Formally Appointed Internal Implementation Leaders | **6** |  |  |  |  |  | ***** |
| Reflecting & Evaluating | **7** |  | ***** |  |  |  |  |

**denotes a strongly distinguishing construct; *denotes a weakly distinguishing construct.

**Consolidated Framework for Implementation Research (CFIR) construct ratings and correlations to fidelity of individual approach to promote smoking cessation change by Primary Health Care (PHC) centre.**

| **PHC centre** | **# of times the construct emerged** | **H** | **D** | **M** | **K** | **B** | **L** | **G** | **A** | **I** | **J** | **Spearman’s correlation** | |
| --- | --- | --- | --- | --- | --- | --- | --- | --- | --- | --- | --- | --- | --- |
| **% of participants for whom the professional has registered to have performed the individual intervention (Very brief or brief intervention + SMS)** |  | **26.6** | **35.9** | **37.7** | **43.6** | **46.8** | **55.2** | **63.4** | **63.6** | **69.4** | **78.4** | **rho** | ***P*** |
| **INTERVENTION CHARACTERISTICS** |  |  |  |  |  |  |  |  |  |  |  |  |  |
| Intervention source | 5 | M | M | I | I | M | I | I | I | M | M | NA | NA |
| Evidence Strength & Quality | 3 | M | M | +1 | M | M | M | -1 | +1 | M | M | 0.000 | 1.000 |
| Relative Advantage | 5 | M | M | -2 | +1 | M | M | -2 | +1 | M | +1 | 0.577 | 0.308 |
| Adaptability | 10 | +1 | -1 | +1 | 0 | 0 | +2 | +1 | +1 | 0 | +1 | 0.267 | 0.562 |
| Complexity | 4 | M | M | M | 0 | -1 | -1 | M | M | -2 | M | -0.775 | 0.225 |
| Design Quality & Packaging | 9 | -1 | +1 | +1 | 0 | M | -1 | -2 | +2 | 0 | 0 | 0.051 | 0.935 |
| **OUTER SETTING** |  |  |  |  |  |  |  |  |  |  |  |  |  |
| Patient Needs & Resources | 7 | +1 | M | M | -1 | 0 | 0 | -1 | -1 | +1 | M | 0.000 | 1.000 |
| Cosmopolitanism | 7 | +2 | -1 | M | +1 | M | M | 0 | -1 | +2 | 0 | 0.000 | 1.000 |
| External Policy & Incentives | 7 | -2 | M | -2 | -2 | -2 | 0 | M | -2 | M | 0 | 1.000 | NA |
| **INNER SETTING** |  |  |  |  |  |  |  |  |  |  |  |  |  |
| Structural Characteristics | 5 | -2 | M | -2 | -1 | -2 | M | M | M | M | 0 | 0.258 | 0.742 |
| Networks & Communications | 8 | +1 | 0 | -1 | 0 | -1 | -1 | M | +1 | M | -2 | -0.463 | 0.355 |
| Culture | 5 | -1 | M | M | +1 | -1 | M | +1 | M | +1 | M | 0.577 | 0.308 |
| Implementation Climate |  |  |  |  |  |  |  |  |  |  |  |  |  |
| Compatibility | 10 | -1 | 0 | +2 | -1 | 0 | -2 | 0 | -2 | +1 | -2 | -0.449 | 0.312 |
| Relative Priority | 6 | -1 | M | M | M | +1 | 0 | M | +1 | +1 | -1 | 0.062 | 0.908 |
| Readiness for Implementation |  |  |  |  |  |  |  |  |  |  |  |  |  |
| Leadership Engagement | 3 | M | -1 | M | M | -2 | M | M | M | M | 0 | 0.500 | 0.667 |
| **Available Resources** | 9 | -2 | M | -2 | -2 | -2 | -2 | +1 | -2 | -1 | -1 | **0.667 | 0.050 |
| **Access to Knowledge & Information** | 9 | -2 | -1 | -1 | M | -1 | -1 | -1 | +1 | 0 | 0 | **0.853 | 0.003 |
| **CHARACTERISTICS OF INDIVIDUALS** |  |  |  |  |  |  |  |  |  |  |  |  |  |
| Knowledge & Beliefs about the Innovation | 10 | -1 | +2 | +1 | +1 | +1 | +1 | -1 | -1 | +1 | 0 | -0.129 | 0.722 |
| Self-efficacy | 7 | +1 | M | -2 | +1 | 0 | -1 | M | -1 | M | -1 | -0.374 | 0.408 |
| Individual Stage of Change | 7 | M | -1 | -1 | M | +1 | -1 | +1 | 0 | M | +2 | 0.601 | 0.115 |
| **PROCESS** |  |  |  |  |  |  |  |  |  |  |  |  |  |
| Planning | 7 | M | -1 | +1 | +1 | M | M | +1 | +1 | 0 | -1 | -0.199 | 0.668 |
| Engaging |  |  |  |  |  |  |  |  |  |  |  |  |  |
| Formally Appointed Internal Implementation Leaders | 6 | -1 | +1 | -1 | -1 | 0 | M | M | +1 | M | M | 0.401 | 0.431 |
| External Change Agents | 7 | +1 | +1 | +1 | +1 | 0 | M | M | +1 | +1 | M | -0.204 | 0.661 |
| Key Stakeholders | 9 | +1 | +1 | M | -1 | 0 | -1 | +1 | +1 | +1 | 0 | 0.203 | 0.601 |
| Innovation Participants | 10 | 0 | +1 | -1 | +1 | +1 | -1 | +1 | 0 | -1 | -2 | -0.308 | 0.386 |
| Executing | 9 | -1 | 0 | +2 | +1 | -1 | M | +1 | +1 | -1 | -1 | -0.549 | 0.159 |
| Reflecting & Evaluating | 7 | +1 | 0 | M | +1 | M | M | +1 | +1 | +2 | -1 | 0.030 | 0.954 |

M, denotes missing data; 0 denotes mixed or neutral data; NA, Not applicable; I, intervention was developed internally

**denotes strongly distinguishing construct: *denotes a weakly distinguishing construct

**Consolidated Framework for Implementation Research (CFIR) construct ratings and correlations to fidelity of individual approach to promote physical activity behaviour change by Primary Health Care (PHC) centre.**

| **PHC centre** | **# of times construct emerged** | **H** | **M** | **L** | **D** | **J** | **B** | **K** | **G** | **I** | **A** | **Spearman’s correlation** | |
| --- | --- | --- | --- | --- | --- | --- | --- | --- | --- | --- | --- | --- | --- |
| **% of participants for whom the professional has registered to have performed the individual intervention (Very brief or brief intervention + SMS)** |  | 22.4 | 41.3 | 44.3 | 49.0 | 63.2 | 64.5 | 65.5 | 68.5 | 70.4 | 75.7 | rho | *P* |
| **INTERVENTION CHARACTERISTICS** |  |  |  |  |  |  |  |  |  |  |  |  |  |
| Intervention source | 5 | M | I | I | M | M | M | I | I | M | I | NA | NA |
| Evidence Strength & Quality | 3 | M | +1 | M | M | M | M | M | -1 | M | +1 | 0.000 | 1.000 |
| Relative Advantage | 5 | M | -2 | M | M | +1 | M | +1 | -2 | M | +1 | 0.289 | 0.638 |
| Adaptability | 10 | +1 | +1 | +2 | -1 | +1 | 0 | 0 | +1 | 0 | +1 | -0.134 | 0.775 |
| Complexity | 4 | M | M | -1 | M | M | -1 | 0 | M | -2 | M | -0.775 | 0.225 |
| Design Quality & Packaging | 9 | -1 | +1 | -1 | +1 | 0 | M | 0 | -2 | 0 | +2 | 0.205 | 0.741 |
| **OUTER SETTING** |  |  |  |  |  |  |  |  |  |  |  |  |  |
| Patient Needs & Resources | 7 | +1 | M | 0 | M | M | 0 | -1 | -1 | +1 | -1 | -0.289 | 0.638 |
| Cosmopolitanism | 7 | +2 | M | M | -1 | 0 | M | +1 | 0 | +2 | -1 | -0.316 | 0.604 |
| External Policy & Incentives | 7 | -2 | -2 | 0 | M | 0 | -2 | -2 | M | M | -2 | 1.000 | NA |
| **INNER SETTING** |  |  |  |  |  |  |  |  |  |  |  |  |  |
| Structural Characteristics | 5 | -2 | -2 | M | M | 0 | -2 | -1 | M | M | M | 0.775 | 0.225 |
| Networks & Communications | 8 | +1 | -1 | -1 | 0 | -2 | -1 | 0 | M | M | +1 | -0.062 | 0.908 |
| **Culture** | 5 | -1 | M | M | M | M | -1 | +1 | +1 | +1 | M | *0.866 | 0.058 |
| Implementation Climate |  |  |  |  |  |  |  |  |  |  |  |  |  |
| Compatibility | 10 | -1 | +2 | -2 | 0 | -2 | 0 | -1 | 0 | +1 | -2 | -0.262 | 0.570 |
| **Relative Priority** | 6 | -1 | M | 0 | M | -1 | +1 | M | M | +1 | +1 | **0.833 | 0.039 |
| Readiness for Implementation |  |  |  |  |  |  |  |  |  |  |  |  |  |
| Leadership Engagement | 3 | M | M | M | -1 | 0 | -2 | M | M | M | M | -0.500 | 0.667 |
| Available Resources | 9 | -2 | -2 | -2 | M | -1 | -2 | -2 | +1 | -1 | -2 | 0.378 | 0.315 |
| **Access to Knowledge & Information** | 9 | -2 | -1 | -1 | -1 | 0 | -1 | M | -1 | 0 | +1 | **0.780 | 0.013 |
| **CHARACTERISTICS OF INDIVIDUALS** |  |  |  |  |  |  |  |  |  |  |  |  |  |
| Knowledge & Beliefs about the Innovation | 10 | -1 | +1 | +1 | +2 | 0 | +1 | +1 | -1 | +1 | -1 | -0.291 | 0.415 |
| Self-efficacy | 7 | +1 | -2 | -1 | M | -1 | 0 | +1 | M | M | -1 | 0.094 | 0.842 |
| Individual Stage of Change | 7 | M | -1 | -1 | -1 | +2 | +1 | M | +1 | M | 0 | 0.551 | 0.157 |
| **PROCESS** |  |  |  |  |  |  |  |  |  |  |  |  |  |
| Planning | 7 | M | +1 | M | -1 | -1 | M | +1 | +1 | 0 | +1 | 0.279 | 0.545 |
| Engaging |  |  |  |  |  |  |  |  |  |  |  |  |  |
| Formally Appointed Internal Implementation Leaders | 6 | -1 | -1 | M | +1 | M | 0 | -1 | M | M | +1 | 0.494 | 0.320 |
| External Change Agents | 7 | +1 | +1 | M | +1 | M | 0 | +1 | M | +1 | +1 | 0.000 | 1.000 |
| Key Stakeholders | 9 | +1 | M | -1 | +1 | 0 | 0 | -1 | +1 | +1 | +1 | 0.526 | 0.146 |
| Innovation Participants | 10 | 0 | -1 | -1 | +1 | -2 | +1 | +1 | +1 | -1 | 0 | 0.361 | 0.306 |
| Executing | 9 | -1 | +2 | M | 0 | -1 | -1 | +1 | +1 | -1 | +1 | -0.268 | 0.521 |
| **Reflecting & Evaluating** | 6 | +1 | M | M | 0 | -1 | M | +1 | +1 | +2 | +1 | *0.759 | 0.080 |

M, denotes missing data; 0 denotes mixed or neutral data; NA, Not applicable; I, intervention was developed internally

**denotes strongly distinguishing construct: *denotes a weakly distinguishing construct

**Consolidated Framework for Implementation Research (CFIR) construct ratings and correlations to fidelity of individual approach to promote dietary behaviour change by Primary Health Care (PHC) centre.**

| **PHC centre** | **# of times the construct emerged** | **H** | **M** | **D** | **J** | **I** | **L** | **K** | **G** | **B** | **A** | **Spearman’s correlation** | |
| --- | --- | --- | --- | --- | --- | --- | --- | --- | --- | --- | --- | --- | --- |
| **% of participants for whom the professional has registered to have performed the individual intervention (Very brief or brief intervention + SMS)** |  | **17.2** | **33.3** | **46.2** | **48.2** | **50.9** | **51.9** | **52.3** | **53.5** | **54.5** | **72.0** | **rho** | ***P*** |
| **INTERVENTION CHARACTERISTICS** |  |  |  |  |  |  |  |  |  |  |  |  |  |
| Intervention source | 5 | M | I | M | M | M | I | I | I | M | I | NA | NA |
| Evidence Strength & Quality | 3 | M | +1 | M | M | M | M | M | -1 | M | +1 | 0.000 | 1.000 |
| Relative Advantage | 5 | M | -2 | M | +1 | M | M | +1 | -2 | M | +1 | 0.289 | 0.638 |
| Adaptability | 10 | +1 | +1 | -1 | +1 | 0 | +2 | 0 | +1 | 0 | +1 | 0.267 | 0.562 |
| Complexity | 4 | M | M | M | M | -2 | -1 | 0 | M | -1 | M | 0.258 | 0.742 |
| Design Quality & Packaging | 9 | -1 | +1 | +1 | 0 | 0 | -1 | 0 | -2 | M | +2 | 0.051 | 0.935 |
| **OUTER SETTING** |  |  |  |  |  |  |  |  |  |  |  |  |  |
| **Patient Needs & Resources** | 7 | +1 | M | M | M | +1 | 0 | -1 | -1 | 0 | -1 | *-0.866 | 0.058 |
| Cosmopolitanism | 7 | +2 | M | -1 | 0 | +2 | M | +1 | 0 | M | -1 | -0.474 | 0.420 |
| External Policy & Incentives | 7 | -2 | -2 | M | 0 | M | 0 | -2 | M | -2 | -2 | 1.000 | NA |
| **INNER SETTING** |  |  |  |  |  |  |  |  |  |  |  |  |  |
| Structural Characteristics | 5 | -2 | -2 | M | 0 | M | M | -1 | M | -2 | M | 0.258 | 0.742 |
| Networks & Communications | 8 | +1 | -1 | 0 | -2 | M | -1 | 0 | M | -1 | +1 | 0.062 | 0.908 |
| Culture | 5 | -1 | M | M | M | +1 | M | +1 | +1 | -1 | M | 0.000 | 1.000 |
| Implementation Climate |  |  |  |  |  |  |  |  |  |  |  |  |  |
| Compatibility | 10 | -1 | +2 | 0 | -2 | +1 | -2 | -1 | 0 | 0 | -2 | -0.468 | 0.290 |
| **Relative Priority** | 6 | -1 | M | M | -1 | +1 | 0 | M | M | +1 | +1 | *0.802 | 0.055 |
| Readiness for Implementation |  |  |  |  |  |  |  |  |  |  |  |  |  |
| Leadership Engagement | 3 | M | M | -1 | 0 | M | M | M | M | -2 | M | -0.500 | 0.667 |
| Available Resources | 9 | -2 | -2 | M | -1 | -1 | -2 | -2 | +1 | -2 | -2 | -0.020 | 0.959 |
| Access to Knowledge & Information | 9 | -2 | -1 | -1 | 0 | 0 | -1 | M | -1 | -1 | +1 | 0.523 | 0.149 |
| **CHARACTERISTICS OF INDIVIDUALS** |  |  |  |  |  |  |  |  |  |  |  |  |  |
| Knowledge & Beliefs about the Innovation | 10 | -1 | +1 | +2 | 0 | +1 | +1 | +1 | -1 | +1 | -1 | -0.291 | 0.415 |
| Self-efficacy | 7 | +1 | -2 | M | -1 | M | -1 | +1 | M | 0 | -1 | 0.037 | 0.937 |
| Individual Stage of Change | 7 | M | -1 | -1 | +2 | M | -1 | M | +1 | +1 | 0 | 0.401 | 0.325 |
| **PROCESS** |  |  |  |  |  |  |  |  |  |  |  |  |  |
| Planning | 7 | M | +1 | -1 | -1 | 0 | M | +1 | +1 | M | +1 | 0.478 | 0.278 |
| Engaging |  |  |  |  |  |  |  |  |  |  |  |  |  |
| Formally Appointed Internal Implementation Leaders | 6 | -1 | -1 | +1 | M | M | M | -1 | M | 0 | +1 | 0.617 | 0.192 |
| External Change Agents | 7 | +1 | +1 | +1 | M | +1 | M | +1 | M | 0 | +1 | -0.408 | 0.363 |
| Key Stakeholders | 9 | +1 | M | +1 | 0 | +1 | -1 | -1 | +1 | 0 | +1 | 0.286 | 0.456 |
| Innovation Participants | 10 | 0 | -1 | +1 | -2 | -1 | -1 | +1 | +1 | +1 | 0 | 0.334 | 0.345 |
| Executing | 9 | -1 | +2 | 0 | -1 | -1 | M | +1 | +1 | -1 | +1 | -0.064 | 0.881 |
| Reflecting & Evaluating | 7 | +1 | M | 0 | -1 | +2 | M | +1 | +1 | M | +1 | 0.516 | 0.295 |

M, denotes missing data; 0 denotes mixed or neutral data; NA, Not applicable; I, intervention was developed internally

**denotes strongly distinguishing construct: *denotes a weakly distinguishing construct

**Consolidated Framework for Implementation Research (CFIR) construct ratings and correlations to fidelity of group approach by Primary Health Care (PHC) centre.**

| **PHC centre** | **# of times the construct emerged** | **M** | **H** | **A** | **I** | **K** | **J** | **G** | **D** | **L** | **B** | **Spearman’s correlation** | |
| --- | --- | --- | --- | --- | --- | --- | --- | --- | --- | --- | --- | --- | --- |
| **% of participants for whom the professional has registered to have recommended the group intervention** |  | **30.3** | **31.4** | **37.6** | **44.0** | **44.0** | **45.4** | **51.4** | **57.5** | **59.7** | **63.5** | **rho** | ***P*** |
| **INTERVENTION CHARACTERISTICS** |  |  |  |  |  |  |  |  |  |  |  |  |  |
| Intervention source | 5 | I | M | I | M | I | M | I | M | I | M | NA | NA |
| Evidence Strength & Quality | 3 | +1 | M | +1 | M | M | M | -1 | M | M | M | -0.866 | 0.333 |
| Relative Advantage | 5 | -2 | M | +1 | M | +1 | +1 | -2 | M | M | M | 0.000 | 1.000 |
| Adaptability | 10 | +1 | +1 | +1 | 0 | 0 | +1 | +1 | -1 | +2 | 0 | 0.134 | 0.775 |
| Complexity | 4 | M | M | M | -2 | 0 | M | M | M | -1 | -1 | 0.258 | 0.742 |
| Design Quality & Packaging | 9 | +1 | -1 | +2 | 0 | 0 | 0 | -2 | +1 | -1 | M | -0.462 | 0.434 |
| **OUTER SETTING** |  |  |  |  |  |  |  |  |  |  |  |  |  |
| Patient Needs & Resources | 7 | M | +1 | -1 | +1 | -1 | M | -1 | M | 0 | 0 | -0.444 | 0.454 |
| Cosmopolitanism | 7 | M | +2 | -1 | +2 | +1 | 0 | 0 | -1 | M | M | -0.406 | 0.498 |
| External Policy & Incentives | 7 | -2 | -2 | -2 | M | -2 | 0 | M | M | 0 | -2 | 1.000 | NA |
| **INNER SETTING** |  |  |  |  |  |  |  |  |  |  |  |  |  |
| Structural Characteristics | 5 | -2 | -2 | M | M | -1 | 0 | M | M | M | -2 | 0.258 | 0.742 |
| Networks & Communications | 8 | -1 | +1 | +1 | M | 0 | -2 | M | 0 | -1 | -1 | -0.370 | 0.470 |
| Culture | 5 | M | -1 | M | +1 | +1 | M | +1 | M | M | -1 | 0.000 | 1.0000 |
| Implementation Climate |  |  |  |  |  |  |  |  |  |  |  |  |  |
| Compatibility | 10 | +2 | -1 | -2 | +1 | -1 | -2 | 0 | 0 | -2 | 0 | -0.633 | 0.127 |
| Relative Priority | 6 | M | -1 | +1 | +1 | M | -1 | M | M | 0 | +1 | 0.247 | 0.637 |
| Readiness for Implementation |  |  |  |  |  |  |  |  |  |  |  |  |  |
| **Leadership Engagement** | 3 | M | M | M | M | M | 0 | M | -1 | M | -2 | **-1.000 | 0.010 |
| Available Resources | 9 | -2 | -2 | -2 | -1 | -2 | -1 | +1 | M | -2 | -2 | 0.260 | 0.499 |
| Access to Knowledge & Information | 9 | -1 | -2 | +1 | 0 | M | 0 | -1 | -1 | -1 | -1 | -0.083 | 0.833 |
| **CHARACTERISTICS OF INDIVIDUALS** |  |  |  |  |  |  |  |  |  |  |  |  |  |
| Knowledge & Beliefs about the Innovation | 10 | +1 | -1 | -1 | +1 | +1 | 0 | -1 | +2 | +1 | +1 | 0.422 | 0.225 |
| Self-efficacy | 7 | -2 | +1 | -1 | M | +1 | -1 | M | M | -1 | 0 | 0.187 | 0.688 |
| Individual Stage of Change | 7 | -1 | M | 0 | M | M | +2 | +1 | -1 | -1 | +1 | 0.050 | 0.906 |
| **PROCESS** |  |  |  |  |  |  |  |  |  |  |  |  |  |
| Planning | 7 | +1 | M | +1 | 0 | +1 | -1 | +1 | -1 | M | M | -0.593 | 0.161 |
| Engaging |  |  |  |  |  |  |  |  |  |  |  |  |  |
| Formally Appointed Internal Implementation Leaders | 6 | -1 | -1 | +1 | M | -1 | M | M | +1 | M | 0 | 0.525 | 0.285 |
| External Change Agents | 7 | +1 | +1 | +1 | +1 | +1 | M | M | +1 | M | 0 | -0.618 | 0.139 |
| Key Stakeholders | 9 | M | +1 | +1 | +1 | -1 | 0 | +1 | +1 | -1 | 0 | 0.106 | 0.785 |
| Innovation Participants | 10 | -1 | 0 | 0 | -1 | +1 | -2 | +1 | +1 | -1 | +1 | 0.079 | 0.828 |
| Executing | 9 | +2 | -1 | +1 | -1 | +1 | -1 | +1 | 0 | M | -1 | -0.604 | 0.113 |
| Reflecting & Evaluating | 7 | M | +1 | +1 | +2 | +1 | -1 | +1 | 0 | M | M | -0.524 | 0.286 |

M, denotes missing data; 0 denotes mixed or neutral data; NA, Not applicable; I, intervention was developed internally

**denotes strongly distinguishing construct: *denotes a weakly distinguishing construct

**Consolidated Framework for Implementation Research (CFIR) construct ratings and correlations to fidelity of community approach by Primary Health Care (PHC) centre.**

| **PHC centre** | **# of times the construct emerged** | **K** | **D** | **L** | **M** | **A** | **B** | **G** | **J** | **H** | **I** | **Spearman’s correlation** | |
| --- | --- | --- | --- | --- | --- | --- | --- | --- | --- | --- | --- | --- | --- |
| **% of participants for whom the professional has registered to have recommended the community intervention** |  | **2.7** | **5.7** | **9.9** | **10.9** | **12.0** | **19.1** | **19.4** | **19.8** | **21.4** | **30.6** | **rho** | ***P*** |
| **INTERVENTION CHARACTERISTICS** |  |  |  |  |  |  |  |  |  |  |  |  |  |
| Intervention source | 5 | I | M | I | I | I | M | I | M | M | M | NA | NA |
| Evidence Strength & Quality | 3 | M | M | M | +1 | +1 | M | -1 | M | M | M | -0.866 | 0.333 |
| Relative Advantage | 5 | +1 | M | M | -2 | +1 | M | -2 | +1 | M | M | 0.000 | 1.000 |
| Adaptability | 10 | 0 | -1 | +2 | +1 | +1 | 0 | +1 | +1 | +1 | 0 | 0.134 | 0.775 |
| Complexity | 4 | 0 | M | -1 | M | M | -1 | M | M | M | -2 | -0.775 | 0.225 |
| Design Quality & Packaging | 9 | 0 | +1 | -1 | +1 | +2 | M | -2 | 0 | -1 | 0 | -0.205 | 0.741 |
| **OUTER SETTING** |  |  |  |  |  |  |  |  |  |  |  |  |  |
| **Patient Needs & Resources** | 7 | -1 | M | 0 | M | -1 | 0 | -1 | M | +1 | +1 | *0.686 | 0.058 |
| Cosmopolitanism | 7 | +1 | -1 | M | M | -1 | M | 0 | 0 | +2 | +2 | 0.632 | 0.252 |
| External Policy & Incentives | 7 | -2 | M | 0 | -2 | -2 | -2 | M | 0 | -2 | M | 1.000 | NA |
| **INNER SETTING** |  |  |  |  |  |  |  |  |  |  |  |  |  |
| Structural Characteristics | 5 | -1 | M | M | -2 | M | -2 | M | 0 | -2 | M | -0.775 | 0.225 |
| Networks & Communications | 8 | 0 | 0 | -1 | -1 | +1 | -1 | M | -2 | +1 | M | 0.123 | 0.816 |
| Culture | 5 | +1 | M | M | M | M | -1 | +1 | M | -1 | +1 | 0.000 | 1.000 |
| Implementation Climate |  |  |  |  |  |  |  |  |  |  |  |  |  |
| Compatibility | 10 | -1 | 0 | -2 | +2 | -2 | 0 | 0 | -2 | -1 | +1 | 0.168 | 0.718 |
| Relative Priority | 6 | M | M | 0 | M | +1 | +1 | M | -1 | -1 | +1 | -0.123 | 0.816 |
| Readiness for Implementation |  |  |  |  |  |  |  |  |  |  |  |  |  |
| Leadership Engagement | 3 | M | -1 | M | M | M | -2 | M | 0 | M | M | 0.500 | 0.667 |
| **Available Resources** | 9 | -2 | M | -2 | -2 | -2 | -2 | +1 | -1 | -2 | -1 | *0.588 | 0.096 |
| Access to Knowledge & Information | 9 | M | -1 | -1 | -1 | +1 | -1 | -1 | 0 | -2 | 0 | 0.128 | 0.742 |
| **CHARACTERISTICS OF INDIVIDUALS** |  |  |  |  |  |  |  |  |  |  |  |  |  |
| Knowledge & Beliefs about the Innovation | 10 | +1 | +2 | +1 | +1 | -1 | +1 | -1 | 0 | -1 | +1 | -0.194 | 0.591 |
| Self-efficacy | 7 | +1 | M | -1 | -2 | -1 | 0 | M | -1 | +1 | M | 0.150 | 0.749 |
| Individual Stage of Change | 7 | M | -1 | -1 | -1 | 0 | +1 | +1 | +2 | M | M | 0.601 | 0.115 |
| **PROCESS** |  |  |  |  |  |  |  |  |  |  |  |  |  |
| Planning | 7 | +1 | -1 | M | +1 | +1 | M | +1 | -1 | M | 0 | -0.299 | 0.515 |
| Engaging |  |  |  |  |  |  |  |  |  |  |  |  |  |
| Formally Appointed Internal Implementation Leaders | 6 | -1 | +1 | M | -1 | +1 | 0 | M | M | -1 | M | -0.031 | 0.954 |
| External Change Agents | 7 | +1 | +1 | M | +1 | +1 | 0 | M | M | +1 | +1 | -0.204 | 0.661 |
| Key Stakeholders | 9 | -1 | +1 | -1 | M | +1 | 0 | +1 | 0 | +1 | +1 | 0.286 | 0.456 |
| Innovation Participants | 10 | +1 | +1 | -1 | -1 | 0 | +1 | +1 | -2 | 0 | -1 | -0.544 | 0.104 |
| Executing | 9 | +1 | 0 | M | +2 | +1 | -1 | +1 | -1 | -1 | -1 | -0.447 | 0.267 |
| Reflecting & Evaluating | 7 | +1 | 0 | M | M | +1 | M | +1 | -1 | +1 | +2 | 0.213 | 0.686 |

M, denotes missing data; 0 denotes mixed or neutral data; NA, Not applicable; I, intervention was developed internally

**denotes strongly distinguishing construct: *denotes a weakly distinguishing construct

**Consolidated Framework for Implementation Research (CFIR) construct ratings and correlations to fidelity of group or community approaches by Primary Health Care (PHC) centre.**

| **PHC centre** | **# of times the construct emerged** | **H** | **M** | **K** | **A** | **J** | **I** | **D** | **G** | **L** | **B** | **Spearman’s correlation** | |
| --- | --- | --- | --- | --- | --- | --- | --- | --- | --- | --- | --- | --- | --- |
| **% of participants for whom the professional has registered to have recommended the group or the community intervention** |  | **33.9** | **34.1** | **39.5** | **43.6** | **48.0** | **52.0** | **57.3** | **57.8** | **61.3** | **65.7** | **rho** | ***P*** |
| **INTERVENTION CHARACTERISTICS** |  |  |  |  |  |  |  |  |  |  |  |  |  |
| Intervention source | 5 | M | I | I | I | M | M | M | I | I | M | NA | NA |
| Evidence Strength & Quality | 3 | M | +1 | M | +1 | M | M | M | -1 | M | M | -0.866 | 0.333 |
| Relative Advantage | 5 | M | -2 | +1 | +1 | +1 | M | M | -2 | M | M | -0.289 | 0.638 |
| Adaptability | 10 | +1 | +1 | 0 | +1 | +1 | 0 | -1 | +1 | +2 | 0 | 0.401 | 0.373 |
| Complexity | 4 | M | M | 0 | M | M | -2 | M | M | -1 | -1 | 0.258 | 0.742 |
| Design Quality & Packaging | 9 | -1 | +1 | 0 | +2 | 0 | 0 | +1 | -2 | -1 | M | -0.718 | 0.172 |
| **OUTER SETTING** |  |  |  |  |  |  |  |  |  |  |  |  |  |
| Patient Needs & Resources | 7 | +1 | M | -1 | -1 | M | +1 | M | -1 | 0 | 0 | 0.000 | 1.000 |
| Cosmopolitanism | 7 | +2 | M | +1 | -1 | 0 | +2 | -1 | 0 | M | M | 0.000 | 1.000 |
| External Policy & Incentives | 7 | -2 | -2 | -2 | -2 | 0 | M | M | M | 0 | -2 | 1.000 | NA |
| **INNER SETTING** |  |  |  |  |  |  |  |  |  |  |  |  |  |
| Structural Characteristics | 5 | -2 | -2 | -1 | M | 0 | M | M | M | M | -2 | -0.775 | 0.225 |
| Networks & Communications | 8 | +1 | -1 | 0 | +1 | -2 | M | 0 | M | -1 | -1 | -0.525 | 0.285 |
| Culture | 5 | -1 | M | +1 | M | M | +1 | M | +1 | M | -1 | -0.289 | 0.638 |
| Implementation Climate |  |  |  |  |  |  |  |  |  |  |  |  |  |
| Compatibility | 10 | -1 | +2 | -1 | -2 | -2 | +1 | 0 | 0 | -2 | 0 | -0.356 | 0.434 |
| Relative Priority | 6 | -1 | M | M | +1 | -1 | +1 | M | M | 0 | +1 | 0.339 | 0.510 |
| Readiness for Implementation |  |  |  |  |  |  |  |  |  |  |  |  |  |
| Leadership Engagement | 3 | M | M | M | M | 0 | M | -1 | M | M | -2 | -0.500 | 0.667 |
| Available Resources | 9 | -2 | -2 | -2 | -2 | -1 | -1 | M | +1 | -2 | -2 | 0.299 | 0.435 |
| Access to Knowledge & Information | 9 | -2 | -1 | M | +1 | 0 | 0 | -1 | -1 | -1 | -1 | 0.101 | 0.796 |
| **CHARACTERISTICS OF INDIVIDUALS** |  |  |  |  |  |  |  |  |  |  |  |  |  |
| Knowledge & Beliefs about the Innovation | 10 | -1 | +1 | +1 | -1 | 0 | +1 | +2 | -1 | +1 | +1 | 0.420 | 0.227 |
| Self-efficacy | 7 | +1 | -2 | +1 | -1 | -1 | M | M | M | -1 | 0 | -0.430 | 0.335 |
| Individual Stage of Change | 7 | M | -1 | M | 0 | +2 | M | -1 | +1 | -1 | +1 | 0.050 | 0.906 |
| **PROCESS** |  |  |  |  |  |  |  |  |  |  |  |  |  |
| Planning | 7 | M | +1 | +1 | +1 | -1 | 0 | -1 | +1 | M | M | -0.359 | 0.430 |
| Engaging |  |  |  |  |  |  |  |  |  |  |  |  |  |
| **Formally Appointed Internal Implementation Leaders** | 6 | -1 | -1 | -1 | +1 | M | M | +1 | M | M | 0 | *0.741 | 0.092 |
| External Change Agents | 7 | +1 | +1 | +1 | +1 | M | +1 | +1 | M | M | 0 | -0.612 | 0.144 |
| Key Stakeholders | 9 | +1 | M | -1 | +1 | 0 | +1 | +1 | +1 | -1 | 0 | 0.194 | 0.618 |
| Innovation Participants | 10 | 0 | -1 | +1 | 0 | -2 | -1 | +1 | +1 | -1 | +1 | -0.243 | 0.499 |
| Executing | 9 | -1 | +2 | +1 | +1 | -1 | -1 | 0 | +1 | M | -1 | -0.536 | 0.171 |
| Reflecting & Evaluating | 7 | +1 | M | +1 | +1 | -1 | +2 | 0 | +1 | M | M | 0.152 | 0.774 |

M, denotes missing data; 0 denotes mixed or neutral data; NA, Not applicable; I, intervention was developed internally

**denotes strongly distinguishing construct: *denotes a weakly distinguishing construct
